# Porous zinc-discs as nanocatalysts for methylene blue dye treatment in water: sensing, adsorption and photocatalytic degradation†

**DOI:** 10.1039/d2ra05245h

**Published:** 2022-12-07

**Authors:** Sarita Devi, Aarushi, Sachin Tyagi

**Affiliations:** CSIR-Central Scientific Instruments Organization, Analytical Techniques Division Chandigarh 160030 India matsachin@gmail.com sachintyagi@csio.res.in +91-172-2657267 +91-172-2642545; Acadamy of Scientific and Innovative Research Chennai India

## Abstract

This paper reports a zinc derived (ZD) porous nanosystem that has been used for selective sensing, adsorption, and photocatalytic degradation of the known hazardous dye, Methylene blue (MB). Using zinc nitrate and 2-aminoterphthalic acid as precursors, the synthesis has been optimized to yield disc-shaped nanoparticles. This luminescent ZD nanoparticles exhibit absorption and emission wavelengths of 328 nm and 427 nm, respectively at an excitation wavelength of 330 nm. In the presence of MB, there is a sharp decrease in the photoluminescence emission intensity of ZD nanoparticles. The detection limit, quenching constant and the binding constant of ZD nanoparticles with MB are found to be 0.31 × 10^−9^ M, 3.30 × 10^6^ M^−1^ and 2.27 × 10^6^ M^−1^ respectively. The impact of contact time, initial MB concentration, and pH on the adsorption process were investigated. The equilibrium data fit well with the Langmuir adsorption isotherm model (*R*^2^ = 0.989) and superlatively fitted to the pseudo-second-order rate model (rate constant: 0.00011 g mg^−1^ min^−1^; adsorption capacity (*q*_e, calc._): 386.1 mg g^−1^; *R*^2^: 0.990). Further, the MB dye degradation was performed under ultra-violet irradiation and ∼95% MB degradation was achieved within 70 min. The experimental data are well fitted to the pseudo-first order kinetics (*R*^2^: 0.99; rate constant: 0.015 min^−1^). These disc shaped ZD nanoparticles can not only facilitate the detection, but also the adsorption and photocatalytic degradation of MB, which can be further processed for environmental remediation applications.

## Abstract 2

Herein, we demonstrate multiutility zinc-based porous disc particles as a nano-catalyst, for highly sensitive detection, adsorption, and photocatalytic degradation of methylene blue up to ∼95% MB within 70 min of ultra-violet irradiation treatment.

## Graphical abstract

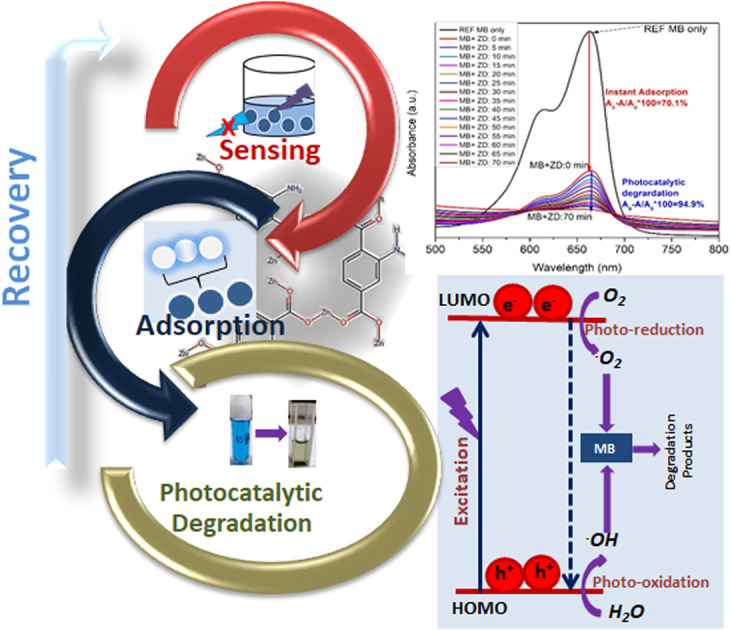

## 1. Introduction

Providing clean water to the population is the biggest challenge these days and the dramatic rise in water contamination due to industrial waste exacerbates the current situation. Synthetic dyes are utilized in a wide range of industries such as pharmaceuticals, cosmetics, food processing, textile, and paint industries.^1^ Wastewater from these industries has a considerable number of dyes which interrupt reoxygenation ability and increases dissolved oxygen demand in aquatic systems.^2^ These organic dyes create severe health issues owing to their carcinogenic, mutagenic, and toxic nature.^3^ Dye degradation requires complex processes and reagents as they are highly stable to light and oxidation reactions; and can be easily accumulated in nature due to their inherent stability.^4^ Methylene blue (MB), (3,7-bis(dimethylamino)-phenothiazin-5-iumchloride), is a basic and cationic dye with aniline moiety and produces greenish-blue colour in water.^5^ This organic dye (C_16_H_18_C_l_N_3_S) is used as an indicator and alkaline stain to observe microscopic life, and as a dying reagent for wool and cotton.^6^ MB is considered as a lethal refractory pollutant generally expelled in textile wastewater.

In recent years, several physical, biological and chemical methods have been employed to abolish dye effluents from water which include conventional adsorption using activated carbon,^7^ biological treatment,^8^ ultrasonic-assisted adsorption,^9^ and photocatalytic degradation.^10^ Adsorption is a widely used facility due to the availability of adsorbents and their ability to be selective to dye molecules along with the potential to scale up for industrial uses. Recently, metal–organic frameworks (MOFs) have been explored as efficacious adsorbents.^11^ MOFs are porous crystalline compounds where organic ligands coordinate with metals and produce interconnecting metal clusters. Such coordination or interconnections in MOFs result in tenability and flexibility to the framework.^12^ MOFs have several advantages over other adsorbents such as modification of pore surfaces, selective adsorptive nature for pollutants, significant porosity, and geometry with tenable pore size and shape.^12^ There are several possible mechanisms which include electrostatic interaction, hydrogen bonding, and π–π interaction between MOF and the pollutants.^13^ These excellent properties render them a unique and worthy candidate for dye adsorption applications. Various MOFs such as N-self-doped TiO_2_/ZrO_2_,^14^ UiO-66 MOFs,^15,16^ porphyrinic Zr-MOF composite^17^*etc.*^18,19^ have been utilized for adsorption of these dyes and other water contaminants.

In the present work, zinc-derived disc-shaped porous material (ZD) exhibiting the same properties as MOFs, has been synthesized and applied for the sensing, adsorption, and photocatalytic degradation of Methylene blue (MB). ZD is prepared using the organic ligand, 2-amino-1,4-benzenedicarboxylic acid as a linker for zinc metal ions. It has been reported that this linker provides the pendant amine groups on the surface of as-synthesized particles.^20^ As-synthesized ZDs are comprised of amino-functionalized pores which provide tenacious frameworks and selectively interact with MB, consequent sensing, and adsorptive removal from contaminated water. Moreover, under UV-irradiation, this ZD serves as a photocatalyst and degrades MB in a very short span.

## 2. Experimental

### 2.1 Synthesis of zinc discs (ZD)

The fast precipitation method was employed for ZD synthesis at RT by following a modified reported procedure.^21^ 4.8 g Zn(NO_3_)_2_·6H_2_O (16 mmol) and 1.32 g 2-aminobenzene dicarboxylic acid (8 mmol) were mixed in 160 mL DMF with continuous stirring at RT. Thereafter, the 9.01 mL of 7.1 M trimethylamine was added gradually to this solution. After the instant formation of white precipitates, this reaction mixture was kept on stirring for 90 min at RT. The solid product was collected by filtration and washed. Washing was done three times with each of the dimethylformamide (DMF) and CH_2_Cl_2_. The filtered product was immersed in 160 mL CH_2_Cl_2_ for 3 days for nucleation.^22^ After the complete nucleation, the product was filtered and dried at 130 °C in a vacuum oven (Navyug India). For further experimentation and characterization, the as-synthesized ZD were suspended on 80 mL DMF and stored at RT in the dark condition.

The procedures of sensing, adsorption, photocatalytic degradation of organic dyes and their selectivity study along with the recovery and reusability are given in ESI data.†

### 2.2 Characterization of synthesized nanomaterial

The emission color of the samples was observed by a UV illuminator (Laby Model-T1-50) at a wavelength of 365 nm. The UV absorption characteristics were monitored for the quantitative as well as qualitative analysis of the materials using the dual-beam (Varian) UV-visible spectrophotometer. The data was cured in the wavelength range 200–800 nm at constant path length in a 3 mL quartz cuvette. The photoluminescence (PL) emission properties were monitored using the Carry Eclipse (Varian) PL spectrophotometer at excitation and emission slit values of 2.5 and 10, respectively in a 3 mL quartz cuvette. The hydrodynamic size and zeta potential studies of the samples at various pH values were performed to predict the dispersity (or agglomeration) of particles by Dynamic Light Scattering (DLS) Zetasizer (ZS90 series). The size and morphological data were acquired by Scanning Electron Microscope (SEM), (Zeiss, QAC Lab, EXPD) and transmission electron microscope (TEM) (TECNAI G2, FEI, USA) respectively. For the SEM characterization, the diluted samples were deposited on ultra-cleaned silicon wafers by drop-casting method and further dried at room temperature (RT). To attain the TEM data, the samples were prepared very carefully on carbon-coated copper grids followed by drying at RT. The functional groups' vibrations were recorded by FTIR (model: Thermo-Scientific Smart Omni-Transmission Nicolet iS10) and Raman spectrophotometers (model: inViaRaman Renishaw). For FTIR analysis, the powder samples were mixed with KBr to form the pellet using a hydraulic pelletizer pump. Raman was performed using the powder material on a glass slide at 785 nm laser and 10% intensity. X-Ray Diffraction (XRD) study was performed by Bruker AXS D8 with Cu-Kα radiation. The absorption and desorption of nitrogen gas were performed during Brunauer–Emmett–Teller (BET) analysis with the help of the Autosorb iQ Station 1/Quantachrome instrument. Before the acquisition of BET data, the powder samples were heat-treated at 120 °C to remove any possible impurities from the pores.

### 2.3 Theoretical assessment of adsorption efficiency of ZD

The experimental adsorption efficiency of ZD is supported with the theoretical assessment. The adsorption capacity (*q*_e_) in mg g^−1^, is the amount adsorbed at equilibrium and can be represented as the eqn (1):1
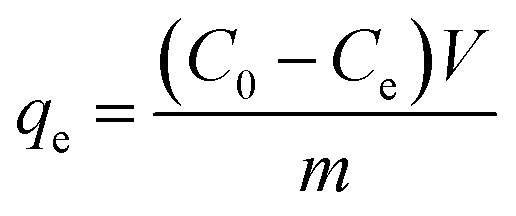


The removal efficiency can be calculated as follows:2
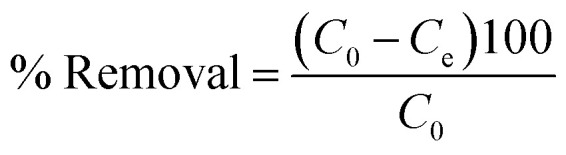


In the eqn (1) and (2), *C*_0_ and *C*_e_ (mg L^−1^) are the initial and final MB concentration at equilibrium, respectively, ‘*q*_e_’ is the adsorption capacity of ZD in mg g^−1^, *V* is the volume of aqueous dye solution (L) and *m* is the amount of adsorbate, ZD (g).

The pseudo-first-order, pseudo-second order, and intraparticle diffusion models are explained for the adsorption mechanism of MB onto the ZD surface. The initial MB concentrations of 10, 20, 40, 60, 80, and 100 mg L^−1^ at pH 8.0 and temperature 298.15 K were studied for the adsorption properties. The first order,^23^ second order,^23^ linear pseudo-second order,^24^ the intraparticle diffusion^25^ and Elovich^23^ kinetic models can be presented as eqn (3), (4), (5), (6) and (7) respectively:3
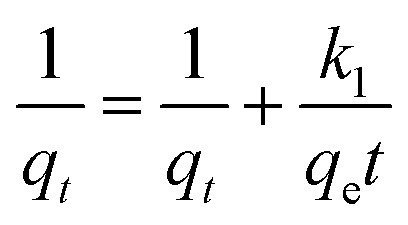
4
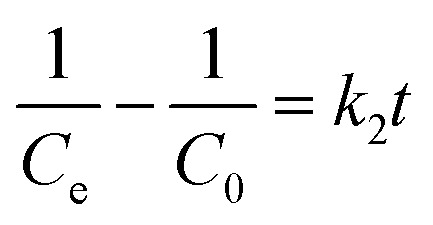
5
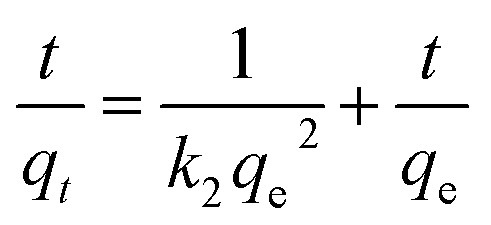
6
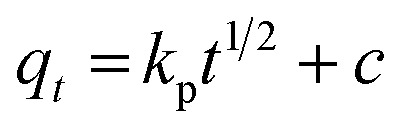
7
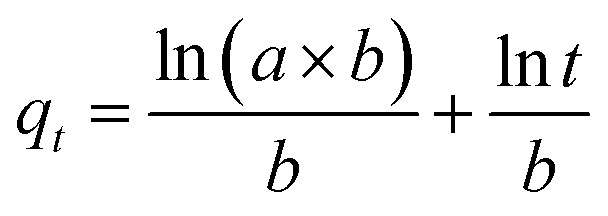
where *q*_e_ (mg g^−1^) is the experimental equilibrium adsorption capacity; *q*_*t*_ (mg g^−1^) is the adsorption capacity at time *t*; *k*_1_ (min^−1^) is the rate constant of the first-order kinetics model; *k*_2_ (g mg^−1^ h^−1^) is the rate constant of the second-order kinetics model; *k*_p_ (mg g^−1^ min^−1/2^) and ‘*c*’ are the rate constant and particle diffusion constant, respectively of the intraparticle diffusion model; but ‘*a*’ (mg g^−1^ min^−1^) and ‘*b*’ (g mg^−1^) in Elovich model represents the rate constant and the rate of adsorption at zero coverage. The values of *k*_1_ and *q*_e_ are found from the slope and intercept values of plots of ln(*q*_e_ − *q*_*t*_) *versus t*, respectively. *k*_2_ is calculated from the plot of *t*/*q*_*t*_ with *t*. *k*_p_ is found from the plot of *q*_*t*_ against *t*_1/2_.

The Langmuir, Freundlich, and Temkin models are being extensively applied to fit the isotherm data. The adsorption isotherm experiments were performed with the initial MB concentration as 10, 20, 40, 60, 80, and 100 mg L^−1^ at 298.15 K to reach adsorption equilibrium. The linear Langmuir,^26^ Freundlich^27^ and Temkin^28^ isotherm models can be represented as following eqn (8), (9), and (10) respectively:8
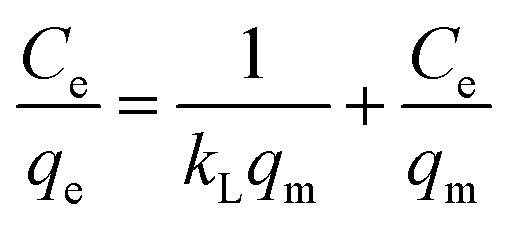
9
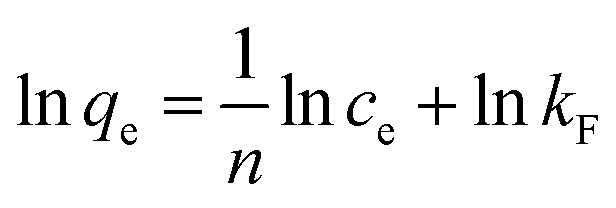
10
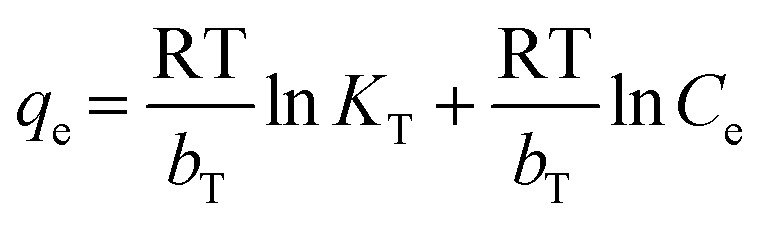


In addition, Langmuir isotherm can also be assessed for a dimensionless constant, the separation factor *R*_L_ as following eqn (11):11
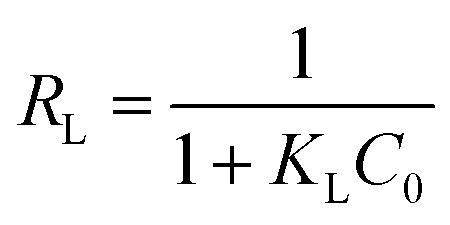
*R*_L_ value points the favourability of isotherms as unfavourable for *R*_L_ > 1, linear for *R*_L_ = 1, and favourable for (0 < *R*_L_ < 1) or an irreversible for *R*_L_ = 0.

## 3. Results and discussion

### 3.1 Characterization of ZD, ZD/MB complex

**Fig. 1:**
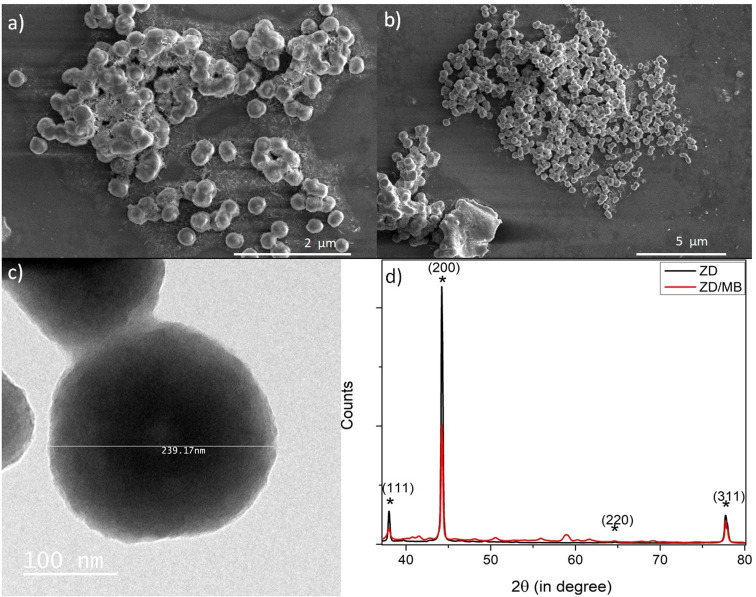
The SEM micrographs of ZD (a) and ZD/MB (b); TEM micrograph of as-synthesized ZD (c); and the XRD spectra of ZD and ZD/MB (d).

The morphological analysis of the as-synthesized particles before and after the MB treatment was performed by SEM and TEM as shown in Fig. 1. The disc-shaped zinc particles were observed from the SEM micrograph as shown in Fig. 1a. The shape and size of the ZD particles after adding to MB were unaltered as shown in Fig. 1b. The TEM micrograph confirms the size of ZD as 239.17 nm (Fig. 1c). The crystal structure of the particles was obtained by the XRD study. The XRD data of the as-synthesized ZD and ZD/MB are shown in Fig. 1d. The Miller indices (*hkl*), *d*-spacing, and FWHM have been calculated for the zinc disc and presented in Table S1.† It can be observed that the Bragg reflection peaks (111), (200), (220), (220), and (311) correspond to the face-centred cubic (FCC) symmetry of ZD following JCPDS-04-0783.^29,30^ Peak positions of ZD remain intact after MB adsorption which describes the stable crystallographic symmetry of ZD without any chemical modification during adsorption. The results show that these structures contain tetrahedral oxygen in chelation with zinc elements.

The spectral data of the hydrodynamic size and zeta potential of the as-synthesized ZD at different pH values is shown and explained in Fig. S1–S3.†

The absorbance and emission characteristics of ZD particles were analysed at various pH to measure their stability and optical performance and shown in Fig. S4 and S5† respectively. The pH of as-synthesized ZD was measured to be 8. In the absorbance spectrum of as-synthesized ZD, the presence of absorbance bands at 230 nm and 328 nm confirms the formation of ZD. Here, the absorbance at the UV region *i.e.*, 230 nm corresponds to the π–π* electronic transaction in aromatic C=C groups. The absorbance band at 328 nm is assigned to the n–π* transitions of C–N groups which confirms the presence of auxochrome, amine groups in the ZD structure.^20^ From Fig. S4a,† it can be observed that with the increase in pH from 2 to 12 there is a hypsochromic and hyperchromic shift in absorbance at higher wavelength *i.e.*, 328–358 nm. The hypsochromic shift of Δ*λ* 30 nm depicts the presence of hydrogen bond energy while the hyperchromic shift represented the increased involvement of lone pair electrons of amine groups in transitions *i.e.*, increased n–π* transitions due to better solvation of electron pairs. It is well noticed from Fig. S4b† that there is no further shift in the absorbance wavelength after pH 6. Therefore, it can be stated that the ZD can deliver constant performance in a wide pH range *i.e.*, from 6 to 12. Thereafter, the emission profiles of these ZD at pH 2, 4, 6, 8, 10, and 12 were monitored by a PL spectrophotometer. The PL spectra of these solutions are presented in Fig. S4c.† It is found that the highest PL emission intensity was delivered by ZD at pH ranging from 4 to 8. At acidic pH 2, the highest emission was observed at *λ*_em_ 453 nm (*λ*_ex_ 370 nm). An increase in pH to 4 resulted in the blue shift of the PL emission wavelength to 433 nm (*λ*_ex_ 340 nm). At higher pH (6–12), there is no such remarkable shift in emission wavelength. The *λ*_em_ remains the same at around 427–425 nm with a fixed excitation wavelength at 330 nm (Fig. S4c†). At pH 12, a further steep decrement is observed in the PL emission intensity of ZD (*λ*_ex_ 330 nm). This trend of variation in Pl emission properties is in complete agreement with that of UV-absorption properties. Therefore, it can be concluded that the ZD is highly stable at wide pH ranges from 4 to 10 except the extreme acidic or basic conditions (Fig. S4d†). The PL emission spectra of ZD at various pHs concerning different excitation wavelengths are presented in Fig. S5.† This optimization study proves the excellent stability of these ZD particles. As found from the DLS, UV-absorbance, and PL data, owing to the high stability of ZD at pH 8, the as-synthesized ZD was preferred to carry out the experimentation, characterizations, and applications. Fig. S6a and b† presents the FT-IR and RAMAN spectra of MB, ZD and ZD/MB complex. BET data were obtained to assess the surface area and porosity of the ZD particles before and after their interaction with MB as shown in Fig. S7a and b† respectively. Both the samples were studied at 77 K with dinitrogen gas. The BET surface area and pore volume measurements are summarized in Tables S2 and S3† respectively. It was observed that the DFT cumulative surface area of ZD decreases from 199.4 m^2^ g^−1^ to 37.03 m^2^ g^−1^ after the treatment with MB, likely due to a highly efficient interaction between ZD and MB and increased pore filling. The DFT pore volume of ZD was observed to decrease from 8.8 × 10^−2^ cm^3^ g^−1^ to 3.49 × 10^−2^ cm^3^ g^−1^ which confirms the successful interaction of MB with ZD particles.

### 3.2 MB detection

**Fig. 2:**
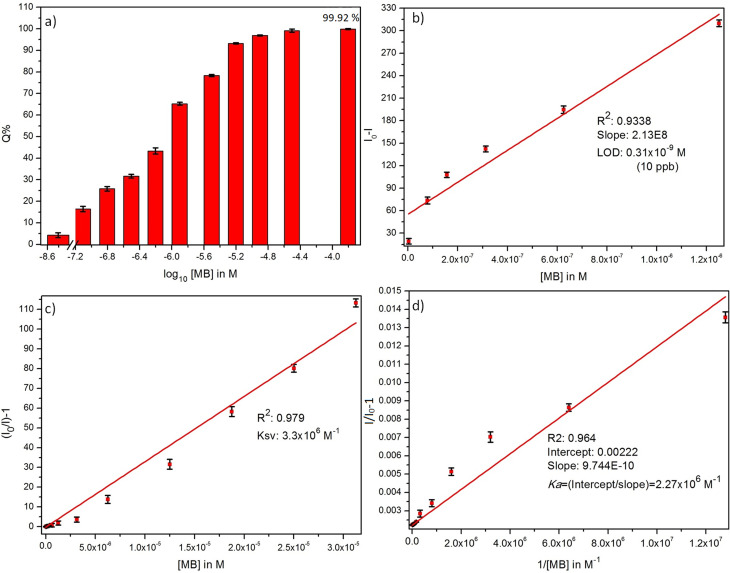
(a) % quenching, (b) detection limit, (c) Stern–Volmer constant, and (d) Benesi–Hildebrand plots of ZD PL emission in presence of MB dye.

The as-synthesized ZD were investigated for any possible interaction with MB dye that might influence their optical properties. Initially, the PL profile of ZD was studied in the presence of MB at emission wavelength 427 nm (*λ*_ex_ 330 nm) as shown in Fig. S8.† It can be observed that with an increase in MB concentration from 3.1 × 10^−9^ M to 1.5 × 10^−4^ M, there is a subsequent decrease in the PL emission intensity of ZD (*n* = 3). This quenching percentage (*Q*%) was calculated using the relation:12
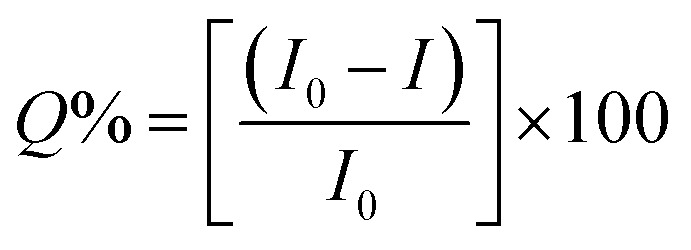
where *I*_0_ and *I* are the initial and final PL emission intensity of ZD in the absence and the presence of MB dye. It was found MB leads in extortionate quenching up to 99.92% at 1.5 × 10^−4^ M concentration (Fig. 2a). Therefore, it can be stated that ZD particles can be utilized successfully for the highly sensitive detection of MB dye in contaminated water.

The theoretical assessment of this proposed sensing material for MB is performed by determining its detection limit (LOD), Stern–Volmer constant (*K*_SV_), and Benesi–Hildebrand association constant (*K*_a_). These parameters are calculated and shown in Fig. 2.

The LOD is measured from the relation:^31^13
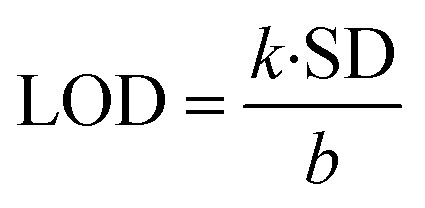
where ‘*k*’ is a constant (*k* = 3), ‘SD’ represents the standard deviation in the variation in PL emission intensity of the blank at 427 nm; and ‘*b*’ stands for the slope of the regression line in the plot of *I*_0_ − *I vs.* [MB] in M. A linear decrease in PL emission intensity of ZD was observed in the presence of MB dye (Fig. 2b). The linear regression relation of this plot (*R*^2^ = 0.934) can be presented as:

14 Δ*I* = *I*_0_ − *I* = 2.13 × 10^8^*C*

Here *C* represents the MB concentration in M. The LOD is achieved to be 0.3 × 10^−9^ M in a broad concentration range of 3.1 × 10^−9^ M to 1.5 × 10^−4^ M MB.

With the optimized conditions, the MB refereed PL quenching in the ZD emission intensity is excellently presented by the Stern–Volmer relation:^32^15
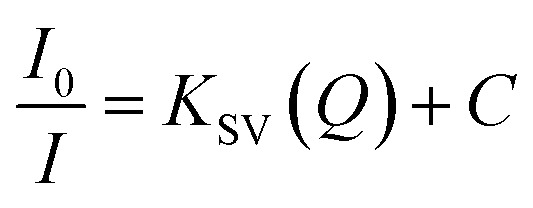
Here, the *I*_0_ and *I* are the initial and final PL emission intensity of ZD in the absence and presence of MB respectively; ‘*K*_SV_’ stands for the Stern–Volmer constant which marks donor/acceptor affinity; ‘*Q*’ represents the MB concentration, and ‘*C*’ is a constant equal to 1. The linear regression equation for the plot of *I*_0_/*I vs.* MB concentration as shown in Fig. 2c can be represented as:16
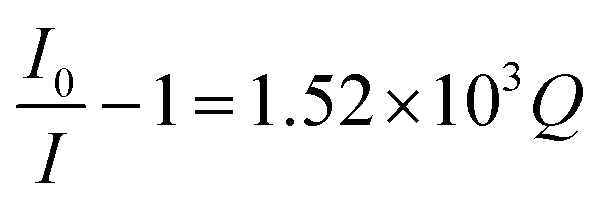


The *K*_SV_ is achieved to be 3.3 × 10^6^ M^−1^ (*R*^2^ = 0.979) in a broad concentration range of 3.1 × 10^−9^ M to 3.1 × 10^−5^ M. The higher value of the quenching constant governs the outrageous affinity of ZD particles for MB dye. Furthermore, it was validated with the measurement of the association constant of these ZD particles with MB. It was calculated by following the Benesi–Hildebrand equation:^33^17
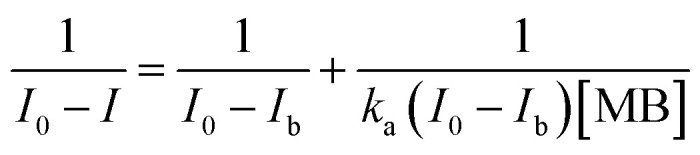
where *k*_a_ stands for the apparent association constant; *I*_0_ and *I*_b_ are the PL emission intensity of ZD particles in the absence and presence of MB dye excess, and ‘*I*’ depicts the observed PL emission intensity of ZD in the presence of different amounts of MB. The plot of 1/(*I*_0_ − *I*) *versus* 1/[MB] is used to find the intercept/slope data which represents the *K*_a_ value (Fig. 2d). The *K*_a_ is achieved to be very high as 2.27 × 10^6^ M^−1^ (*R*^2^: 0.964). The elevated values of *K*_SV_ and *K*_a_ witness the ultrahigh sensitivity of ZD for MB. This theoretical assessment of the ZD particles-based luminescent sensing probe for MB detection validates its ultra-sensitivity for the dye. Therefore, it can be concluded that ZD particles can be successfully utilized for –instant and highly accurate sensing of MB in contaminated water streams before its consumption. The selectivity assessment of ZD for MB over other dyes is performed and presented in Fig. S9.†

### 3.3 Adsorption of MB

**Fig. 3:**
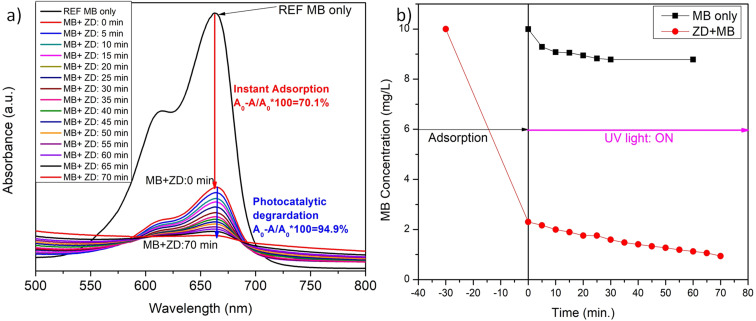
The variation in the (a) absorbance intensity of ZD in the presence of MB and (b) final concentration of MB after its instant addition in ZD and concerning the UV-irradiation time.

The impact of 10 mg L^−1^ ZD on the concentration of 10 mg L^−1^ MB in aqueous solution at 298 K was read and depicted in Fig. 3. The instant, rapid and visible adsorption of MB was noticed up to 70% with this high concentration of adsorbent. Thereafter, the adsorption slows due to the saturation of ZD with MB, and equilibrium was attained. In Fig. 3a, it can be observed that after the instant adsorption of MB, the effect of UV light is examined on the ZD/MB from the irradiation time of 0 min to 70 min at an interval of 5 minutes to find the photocatalytic efficiency of ZD. The steep decrease in the MB concentration can be noticed in the presence of ZD (Fig. 3b).

The effect of contact time and initial dye concentration are two important criteria for determining the rate of dye absorption, the time required to attain the equilibrium by adsorbent–adsorbate system, and the adsorption capacity of the adsorbent. To study the impact of agitation time on the ZD adsorption efficiency, the 0.025 mg L^−1^ ZD was added to 10 mg L^−1^ MB (Fig. S10†). It can be observed that the equilibrium was attained after 100 minutes at this low concentration of adsorbent (Fig. S10a†). After 100 minutes, an unremarkable variation in the absorption intensity of MB solution was noticed (Fig. S10b†). At equilibrium, approximately 88.5% of the initial MB concentration was adsorbed (Fig. S10c†). Therefore, it can be stated that at 0.025 mg L^−1^ concentration, ZD delivered the 88.5% removal efficiency for MB in the solution. The observed adsorption capacity, experimental (mg g^−1^) of ZD was also calculated and shown in Fig. S10d.†

**Fig. 4:**
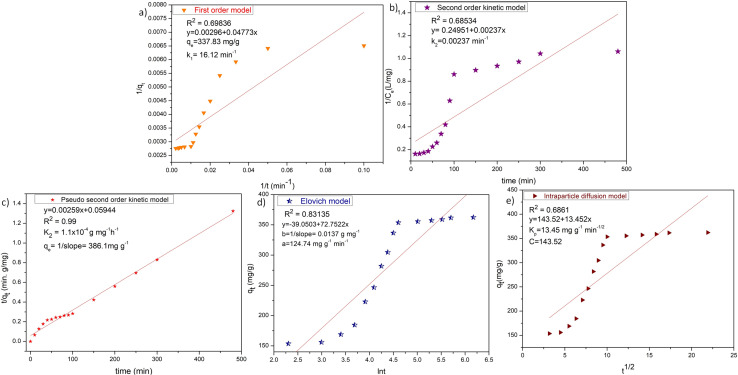
(a) First-order kinetic, (b) second-order kinetic, (c) pseudo-second order kinetic, (d) Elovich and (e) intraparticle diffusion models for ZD-mediated adsorption of MB.

The experimental was achieved up to 353.5 mg g^−1^ in the presence of 0.025 mg L^−1^ ZD adsorbent. After increasing the contact time further, the qe stays nearly constant. The rate constant for the adsorption was calculated by following the first-order kinetic, second-order kinetic, pseudo-second kinetic, Elovich model, and diffusion models. Fig. 4 and Table S4† summarize the linear relationships and the findings of the fittings of the experimental data to these kinetic models for MB adsorption onto ZD.

The low correlation coefficient of the Elovich model plot (*R*^2^ = 0.83135), confers that the experimental data does not fit this model. Therefore, the Elovich model is not valid for this ZD/MB system. Although the calculated *q*_e_ value of the first-order kinetic (337.83 mg g^−1^) aligns with the experimental *q*_e_ value (353.5 mg g^−1^) but the very low value of *R*^2^ = 0.69836 suggests that the first-order kinetic model is also not obeyed by ZD/MB system. Similarly, the second-order kinetic is also not the appropriate model for the adsorption of MB on ZD due to the very low *R*^2^ = 0.68534 in this model. Contrary, the pseudo-second-order model is well followed than other kinetic models with *R*^2^ = 0.99. Besides, the calculated *q*_e_ value (386.1 mg g^−1^) from the pseudo-second-order model is also in great agreement with that of the experimental *q*_e_ (353.5 mg g^−1^) value. This data strongly recommends that the ZD/MB-based adsorption system obeys the pseudo-second-order kinetic model.

It can be concluded that the adsorption immensity might be driven by the higher driving forces resulting in the fast transfer of MB molecules to the ZD surface; the obtainability of the uncovered surface area of ZD; and finally, by the existing unoccupied active sites on the ZD adsorbent.^34^

Proceeding to the diffusion model, intraparticle diffusion is a preliminary phase followed by the final adsorption of any molecule onto the adsorbent in such systems. Additionally, intraparticle diffusion can act as a rate-limiting step for the adsorption in our ZD/MB system.^35^ Therefore, the intraparticle diffusion model was also examined to decide the underlying diffusion mechanism for this system. Since, if intraparticle diffusion is the sole rate-limiting mechanism governing the adsorption process, then the plot *q*_*t*_ against *t*_1/2_ should be linear and pass through the origin.^36^ Otherwise, any additional process is also accounted for in net adsorption.^37^ The intraparticle diffusion model plot is shown in Fig. 4e which has a low value of regression coefficient (*R*^2^ = 0.6861) and intercept ≠ 0. Therefore, it is depicted that the intraparticle diffusion was not the sole rate-limiting step and some other diffusion force is also simultaneously involved in the reaction kinetics regulation.

**Fig. 5:**
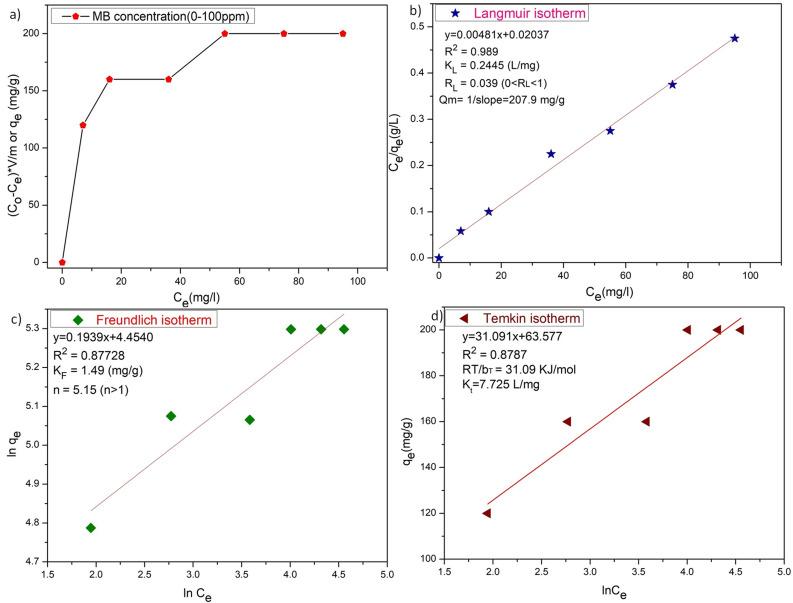
(a) The plot of adsorption capacities *versus* initial concentration of MB, (b) Langmuir isotherm, (c) Freundlich isotherm, and (d) Temkin isotherm for ZD-mediated adsorption of MB.

The adsorption isotherms for the ZD/MB adsorption system with the initial dye concentration of 10, 20, 40, 60, 80, and 100 mg L^−1^ were also determined (Fig. 5). In Fig. 5a, it is well clear that the ZD attains saturation in the presence of 60 mg L^−1^ initial MB concentration in solution. The linear forms of the Langmuir, Freundlich and Temkin isotherms for the adsorption of these MB concentrations onto ZD are shown in Fig. 5b, c, and d respectively. The isotherm equations and the various parameters for each isotherm produced from their slope and intercept values are dictated in Table S5†.^38^ The higher correlation coefficient value quantifies the isotherm's fitness to the experimental data and determines the best-fit isotherm. It can be noted that the equilibrium data fit well to the Langmuir adsorption isotherm model with a highest *R*^2^ value (0.989) than that of the Freundlich and Temkin adsorption isotherm models with lower correlation coefficients (*i.e.*, 0.87728 and 0.8787 respectively). Usually, the value of *n* in Freundlich isotherm, being greater than unity (*n* > 1) signifies that an adsorbate is affirmatively adsorbed on the adsorbent. In this study, *n* is highly exceeding than one (*n* = 5.15) proving that ZD is a pertinent adsorbent for MB adsorption from an aqueous solution.^38^ Furthermore, the *R*_L_ value, a critical parameter of the Langmuir isotherm, lies between 0 and 1 (*i.e.*, 0.039). Therefore, *R*_L_ value also well aligns with the favourability of the adsorption process. The comparison of the maximum uptake/adsorption of MB onto ZD with that of various other reported adsorbents including MOFs is presented in Table S6.†

### 3.4 Photocatalytic degradation of MB by ZD

The photo-degradation activity of MB dye was executed to assess the photocatalytic efficiency of ZD nanoparticles at pH 8. For reference purposes, only MB solution (without ZD) was irradiated under UV-illumination for 1 hour at an interval of 5 min (Fig. S11†) and discussed. Fig. S12a† illustrates that in the absence of ZD catalyst; only about 13% MB degradation was possible even under UV irradiation. To monitor the effect of the presence of ZD photocatalyst in an exact concentration of MB solution (10 mg L^−1^), initially 10 mg L^−1^ of ZD was added to it and exposed to UV irradiation for 70 minutes (Fig. 3). The variation in the absorbance intensity of MB is calculated for the relation:18
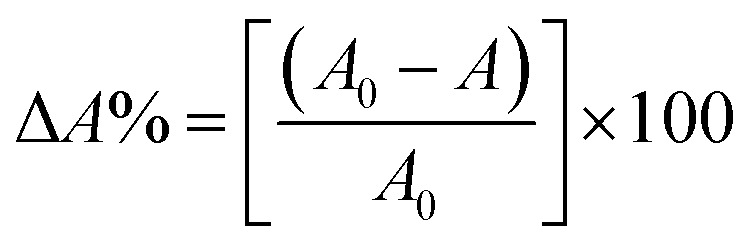
Here, *A*_0_ and *A* are the absorption intensities of MB in the absence and the presence of ZD under UV-irradiation at *λ*_abs_ 663 nm. At the adsorption–desorption equilibrium as achieved under dark conditions (Fig. 3a), approximately a 70% decrease in the absorption intensity was witnessed. Thereafter, the sample was irradiated with UV-light to achieve the complete degradation of MB. After 70 minutes of UV-irradiation, an approximately 95% decrease in absorption intensity has been achieved which is commendably higher (6×) than that of only MB under UV-irradiation (∼13%) (Fig. S12a†). The FWHM of the absorption peaks at wavelength 663 nm of ZD/MB irradiated with UV-light was increased about two times *i.e.*, from 60 to 120 which is comparatively higher than only MB under UV light (Fig. S12b†). The steep drop in the MB concentration from its initial concentration (10 mg L^−1^) in the presence of ZD-nanocatalyst under UV-irradiation can be witnessed in Fig. 3b. It can be noticed that under UV-light treatment ZD is performed as a photocatalyst in the degradation of MB as there is no such effect of this irradiation on the concentration of MB in absence of ZD in solution.

Furthermore, the effect of photocatalyst dosage on MB removal efficiencies under UV-vis irradiation was also examined by varying the ZD concentration in 10 mg L^−1^ MB solution to 10 mg L^−1^ and 20 mg L^−1^. Fig. S13a† demonstrates the relative change in dye concentration (*C*_*t*_/*C*_0_) with the reaction time at different dosages of ZD. Here *C*_0_ is the MB concentration at *t* = 0 min of UV-irradiation after attaining the adsorption–desorption equilibrium, and *C*_*t*_ is the MB concentration under UV exposure at a specific time from 5 min up to 70 min. It can be depicted from Fig. S13a† that with the increase in ZD catalyst concentration, there is a direct enhancement in the degradation of MB in the solution. It happens due to the increased amount of adsorbed MB owing to the increasing active sites and additional surface area of the photocatalyst. At time *t*, a lesser amount of MB is present in the solution containing the 20 mg L^−1^ ZD in comparison to the 10 mg L^−1^ ZD.

The percentage decrease in the MB concentration (removal efficiency) in the respective samples is measured from these *C*_0_ and *C*_*t*_ data by following the equation:19
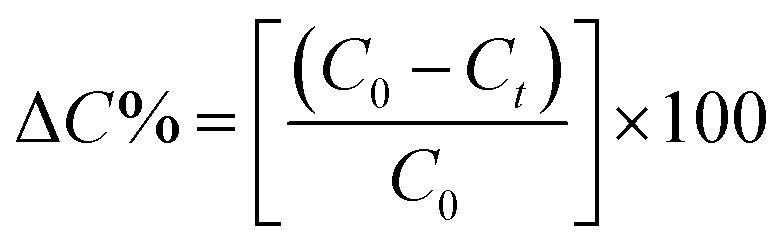


It is well versed that with the increment in photocatalyst dosage, higher MB degradation efficiencies are achieved. The MB removal efficiencies of 10 mg L^−1^ and 20 mg L^−1^ ZD at the same time of UV-irradiation *i.e.*, 70 minutes are found to be 68% and 80%, respectively (Fig. S13b†). Although beyond this concentration of ZD, the MB removal efficiency was reduced. It might be due to the increased physio-chemical interactions of the MB with ZD and its surface coverage. ZD particles could have aggregated into the clusters after achieving the optimum dose and consequently hindering the UV light from interacting with the active surface of ZD. Ultimately, it might have reduced the –OH generation. Therefore, it can be stated that a dose of 20 mg L^−1^ ZD catalyst could be optimum for MB degradation under UV-treatment. This finding implies that UV-light played an important part in MB removal by ZD. The colorimetric change in MB dye solution irradiated with UV-light in the presence and the absence of ZD catalyst is represented in the pictures shown in Fig. S14 and S15,† respectively.

**Fig. 6:**
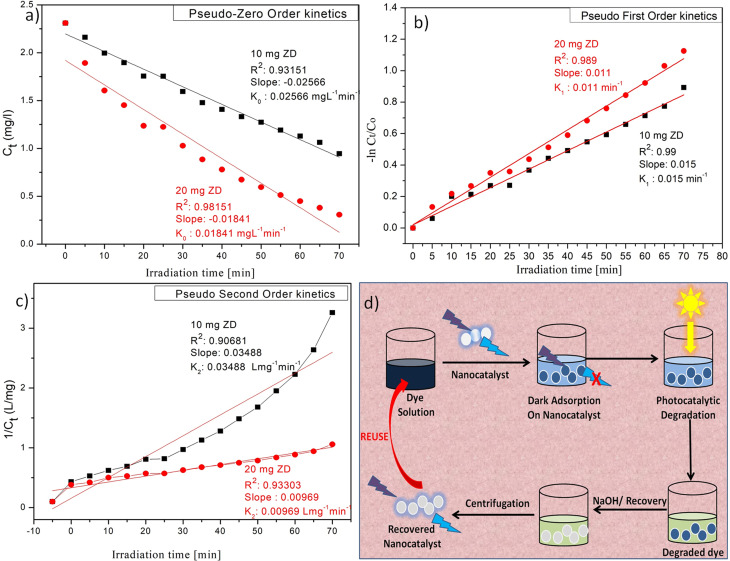
(a) Pseudo-zero order, (b) pseudo-first order, (c) pseudo-second-order kinetic models for ZD-mediated photocatalytic degradation of MB; and (d) the graphical overview of the study.

Photocatalytic degradation kinetics is evaluated according to the pseudo-zero, pseudo-first, and pseudo-second-order kinetic models (Fig. 6) using the eqn (20), (21) and (22) respectively:^39^

20 *C*_*t*_ = *C*_0_ − *k*_0_*t*

21
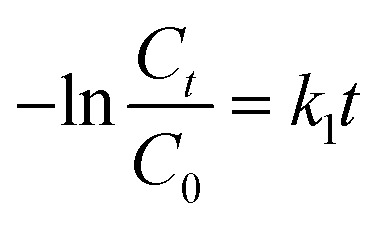
22
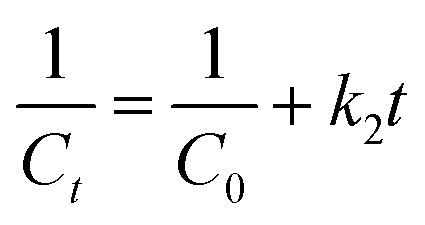
where *C*_0_ and *C*_*t*_ are the termed as the concentrations of MB dye (mg L^−1^) in ZD aqueous solution at time *t* = 0 after achieving the adsorption equilibrium and at a time ‘*t*’ of UV irradiation, respectively; and *k*_0_ is the pseudo-zero-order rate constant (mg L^−1^ min^−1^); *k*_1_ is the pseudo-first-order rate constant (min^−1^) and *k*_2_ is the pseudo-second-order rate constant (L mg^−1^ min^−1^) and *t* is the time (min). The correlation coefficient values and rate constants of these kinetic models for 10 mg L^−1^ and 20 mg L^−1^ ZD dosage are summarized in Table S7.† It can be stated that the experimental data of photocatalytic degradation of MB does not have a good fit in pseudo-zero and pseudo-second-order kinetic models (Fig. 6a and c) with the low values of *R*^2^ (0.931 for 10 mg L^−1^ ZD and 0.981 for 20 mg L^−1^ ZD) and (0.906 for 10 mg L^−1^ ZD and 0.933 for 20 mg L^−1^ ZD) respectively. Whereas the photocatalytic degradation of MB pollutant under UV treatment well obeys the pseudo-first-order kinetic model with the high correlation coefficient values, *R*^2^ = 0.99 and *R*^2^ = 0.989 in the presence of 10 mg L^−1^ and 20 mg L^−1^ ZD dosage, respectively (Fig. 6b). The rate constants of pseudo-first-order reaction are found to be 1.5 × 10^−2^ min^−1^ and 1.1 × 10^−2^ min^−1^ with 10 mg L^−1^ and 20 mg L^−1^ ZD nanoparticles. The overall study is compiled and shown as schematic in Fig. 6d. The selectivity study is presented in Fig. S16.† The mechanisms of adsorption, photocatalytic degradation and pH-mediated behaviour are represented in Fig. S17, S18 and S20† respectively. The recovery and reusability parameters are summarized in Table S8† and shown in Fig. S19 and S21.† This research work is compiled in Fig. S22.†

## 4. Conclusions

The current work demonstrates that the zinc-disc material (ZD) was successfully fabricated and employed for the first time in selective sensing, adsorption, and photocatalytic degradation of the cationic dye, MB. The PL emission intensity of as-synthesized ZD decreases in the presence of MB dye and reaches a detection limit of 0.31 × 10^−9^ M. The data shows that the maximum equilibrium adsorption capacity of ZD for MB dye is (*q*_e, calc._): 386.1 mg g^−1^ and selective concerning other dyes. It was found that the Langmuir model is a better fit for the adsorption of MB to ZD and followed pseudo-second-order kinetics. The computed *q*_e_ values correspond well with the actual values at given MB concentrations with high regression coefficients. Moreover, the presence of free electrons and holes during UV irradiation results in the degradation of MB dye. The photocatalytic degradation of MB pollutant under UV treatment obeys well the pseudo-first-order kinetic model with the high correlation coefficient values, *R*^2^ = 0.99 in the presence of 10 mg L^−1^ ZD dosage with rate constant of 1.5 × 10^−2^ min^−1^. Therefore, it can be summarized that under the optimized conditions (10 mg L^−1^ of ZD, pH 8, and 10 mg L^−1^ of initial MB concentration) an almost complete MB degradation can be attained within 70 minutes of UV-irradiation. The single luminescent and stable porous ZD are successfully utilized for highly sensitive detection of MB, its instant adsorption, and photocatalytic degradation within an hour which is comparatively very fast and efficient than previously reported materials. These findings suggest the good applicability of this material for various applications such as wastewater treatment, filtration-membrane fabrication, biosensing, and catalytic degradation of organic lethal components.

## Author contributions

Sarita Devi: conceptualization, methodology, writing—original draft preparation, data curation, project administration, funding acquisition; Aarushi: conceptualization, writing and editing, data curation, Sachin Tyagi: supervision, reviewing, and editing.

## Conflicts of interest

Authors have no competing interests to declare.

## Supplementary Material

RA-012-D2RA05245H-s001
