# Supplementary material for: Porous zinc-discs as nanocatalysts for methylene blue dye treatment in water: sensing, adsorption and photocatalytic degradation
Source: RSC Adv. 2022 Dec 7;12(54):34951–61. doi: 10.1039/d2ra05245h (PMC9728022; doi:10.1039/d2ra05245h)
Supplement: RA-012-D2RA05245H-s001 [file RA-012-D2RA05245H-s001.pdf]

Electronic Supplementary Material (ESI) for RSC Advances.
This journal is © The Royal Society of Chemistry 2022

Porous Zinc-Discs as Nanocatalyst for Methylene Blue Dye Treatment in Water: Sensing, Adsorption and Photocatalytic Degradation

[1]Sarita Devi, [1,2]Aarushi and [1,2]Sachin Tyagi*

[1]CSIR-Central Scientific Instruments Organization, Chandigarh, INDIA

[2]Acadamy of Scientific and Innovative Research, Chennai, INDIA

*Corresponding Author Postal Address: Analytical Techniques Division, CSIR-CSIO, Chandigarh 160030, India Tel.: +91-172-2642545 (O); Fax: + 91-172-2657267

E-mail: matsachin@gmail.com, sachintyagi@csio.res.in (Dr. SachinTyagi)

1. Materials and methods

Zinc nitrate hexahydrate [$(ZnNO_3)_2.6H_2O$)] (Molychem), 2-aminoterphthalic acid (H_2ATA) (Alfa Aesar), triethylamine (TEA) (Merck), dichloromethane (CH_2Cl_2) (Rankem), Sodium hydroxide (NaOH) pellets (Rankem), ethanol, acetone, dimethylformamide (DMF) (Emplura) andHCl (Rankem) were of analytical grades. Indigo Carmine (IC) ($C_{16}H_8N_20_8S_2$; Mol.wt. 466.36 g/mol; Sisco Research Laboratories Pvt. Ltd.), Methylene blue trihydrate (MB) ($C_{16}H_{18}ClN_3S.H_2O$; Mol.wt. 319.85 g/mol; SRL), Crystal Violet (CV) ($C_{25}H_{30}N_3Cl$; Mol.wt. 407.979 g/mol; SRL), Trypan blue (TB) ($_{C34}H_{24}N_6Na_40_{14}S_4$; Mol.wt. 872.88 g/mol; SRL), Rhodamine B (RhB) ($C_{28}H_{31}ClN_2O_3$; Mol.wt. 479.02 g/mol. All the chemicals and reagents were applied without any further purification. Double distilled water (DI H_2O) with a resistivity of 18 MΩcm was used in all experiments as it was received from the Millipore purification system.

1.1 Sensing of organic dyes and selectivity study

The sensing study of dye was performed using PL spectroscopy. An aqueous stock solution (1000 ppm) of MB, RhB, TB, and IC was prepared in DI water. 1 g of respective dye was dissolved in 1 L DI water to generate the stock solution. A series of aqueous solutions with a distinct concentration of MB dye were prepared from the stock solution. To determine the surface charge of the as-prepared0.1 mg/ml ZD samples, the pH 2-12 was adjusted using 0.1 M HCl and 0.1 M NaOH solutions. The effect of pH (2-12) on the absorbance and Pl emission properties of as-synthesized ZD were also monitored. The interaction of the dye with ZD was studied at selected pH (i.e.,2, 8, and 12). MB sensing study was performed using the 0.1mg/ml ZD solution as a reference. MB was added into this ZD solution to have its final concentration varying as$3.1x10^{-9}$ M, $7.8x10^{-8}$ M, $1.5x10^{-7}$ M, $3.1x10^{-7}$ M, $6.2x10^{-7}$ M, $1.2x10^{-6}$ M, $1.8x10^{-6}$M, $2.5x10^{-6}$ M, $3.1x10^{-6}$ M, $6.2x10^{-6}$ M, $1.2x10^{-5}$ M, $1.8x10^{-5}$ M, $2.5x10^{-5}$ M, $3.1x10^{-5}$ M to $1.5x10^{-4}$ M in the respective sample. The variation in the emission intensity of the reference ZD sample was monitored with PL spectroscopy.

1.2 Adsorption of Organic dyes and selectivity study

Before the adsorption process, the adsorbent or ZD was dried under vacuum at 80°C and stored in the desiccators. Initially, the 10mg/L ZD was used as a reference and studied for the adsorption of 10 mg/L MB in solution. The impact of contact time on the adsorption capacity (q_e) of ZD (0.025 mg/L) for MB removal (10 mg/L) was read by varying the contact time from 0 to 480 minutes. Further, the best fitted reaction-order kinetic was also determined. The effect of initial adsorbate concentration on

the adsorption efficiency of ZD was studied at 10, 20, 40, 60, 80, and 100 mg/L concentrations of MB in solution. This study was utilized to obtain the adsorption isotherm parameters for this ZD/MB-based adsorption system.

The adsorption experiments were followed through in a 100 ml flask at 25°C. The ZD and MB solutions were mixed well with magnetic stirring at 120 rpm for a specific time interval of contact. After a predetermined time, the reaction mixtures were centrifuged and the adsorbate concentration in the solution was analyzed using a UV-Vis spectrophotometer to find the adsorption capacity and removal efficiency of ZD. To determine the effect of various pHs on the adsorption capability, the pH of ZD was pre-adjusted with 0.1M NaOH and 0.1 M HCl.

1.3 Photocatalytic degradation of dye

The photocatalytic performance of ZD nanocatalyst was monitored by degrading MB dye and verified with UV-Vis absorption spectroscopy. Under the dark conditions, the 10 mg/L ZD was used as a reference and studied for the photocatalytic degradation of 10 mg/L MB at ambient temperature. Initially, the solution was stirred to attain the adsorption-desorption equilibrium in a 500 ml capacity reactor. Similarly, IC, Rh B, and CV dye solutions were also prepared with the ZD in dark. Thereafter, the photocatalytic reaction of this solution was performed under a 400W metal halide arc lamp. The experimental parameters such as photocatalyst dosage (10 mg/L and 20mg/L ZD) and reaction time were optimized to facilitate the MB removal from the samples. The variation in absorption profile of the respective solutions is monitored and recorded at an increased time interval of 5 min up to 70 minutes. Before recording the data, the treated samples were collected by centrifugation of the samples at 10,000 rpm. Finally, the reaction kinetics along with their respective rate constants and correlation coefficients were calculated. Besides, the selective degradation efficiency of ZD for MB under UV-irradiation was also studied with the different dyes such as IC, RhB, and CV at concentrations as 7.8x10^{-8} M, 6.2x10^{-7} M, and 6.2x 10^{-6} M. The before and after treatment effect of ZD nanocatalyst on the MB concentrations were determined using a UV–Vis spectrophotometer at 663 nm of wavelength.

1.4 Recovery and Reusability

The ZD was recovered from ZD/dye complex by applying the physical and chemical treatments and compared for the effectiveness of the utilized method. Physically, the ZD/dye complexes were subjected to ultrasonic treatment to split the physical/weak interactions between the adsorbed dye molecules from the ZD surface. Chemically, the ZD/dye complex was treated with 5M NaOH to recover the ZD nanocatalyst. The absorption intensity of the ZD/dye complex was assessed before and after the respective treatment to evaluate their efficiency. Thereby, the recovered ZD was reused for 3 cycles to adsorb the dye and evaluated the efficiency using the UV-absorbance data.

2. Result and Discussion

2.1 Hydrodynamic size and Surface potential at pH

Figure S1 and figure S2 show the spectral data of the hydrodynamic size and zeta potential, respectively of the as-synthesized ZD at different pH values. It can be observed that with the increase in pH from 2 to 12, there is a decrease in the hydrodynamic size of ZD (Fig S3a). It can be credited to the possible involvement of –COOH groups of ZD to form $–COO^-$ at higher pH which might have resulted in the distortion of superficial layers of ZD up to a margin. The zeta potential of ZD is pH-dependent and can be attributed to the existence of the $–NH_2$ group at ZD (Fig. S3b). At pH 2, a highly positive charge, +28.4 mV, is acquired by ZD due to the presence of excessive H^+ ions in the solvent which induces the formation of $-NH_3^+$ of ZD. With an increase in pH

from 4 to 12, the negative surface charge indicates the removal of hydrogen ions owing to the presence of OH- ions in the solvent. It tends to form a stern layer and a slipping plane around the dispersed ZD particles. The magnitude of the zeta potential shows the degree of electrostatic repulsion between adjacent, similarly charged particles. Therefore, the ZD particles are well stable in dispersion at pH 8-10. The results indicate that NH_2-containing ZD could easily adsorb and interact with cationic pollutants, MB, at this pH range of 8-10 through electrostatic attraction. Beyond pH 10, there could be elevated alkali-mediated interference in MB structure which had resulted in its degradation at pH12 [1].

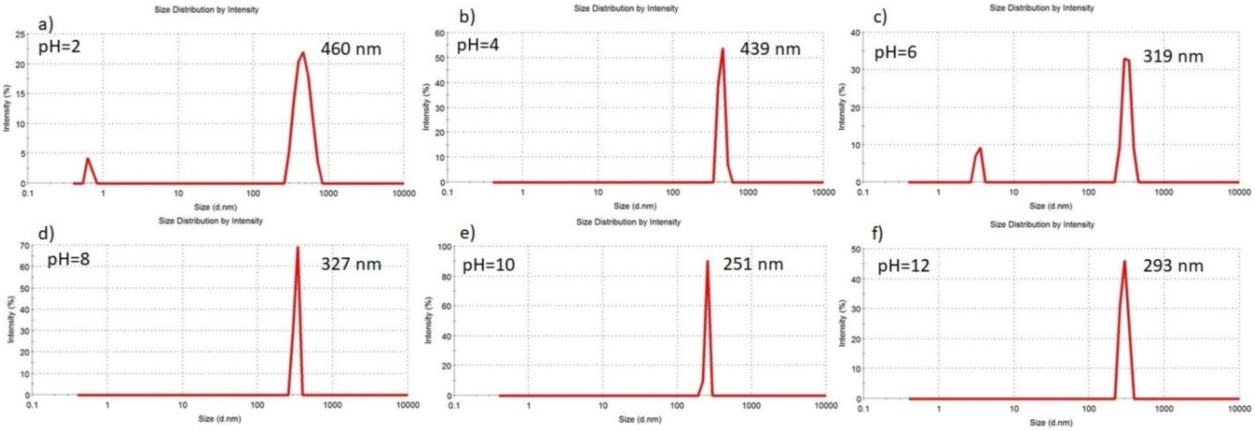

Fig. S1: The hydrodynamic size of as-synthesized ZD at pH 2, 4, 6, 8, 10, and 12.

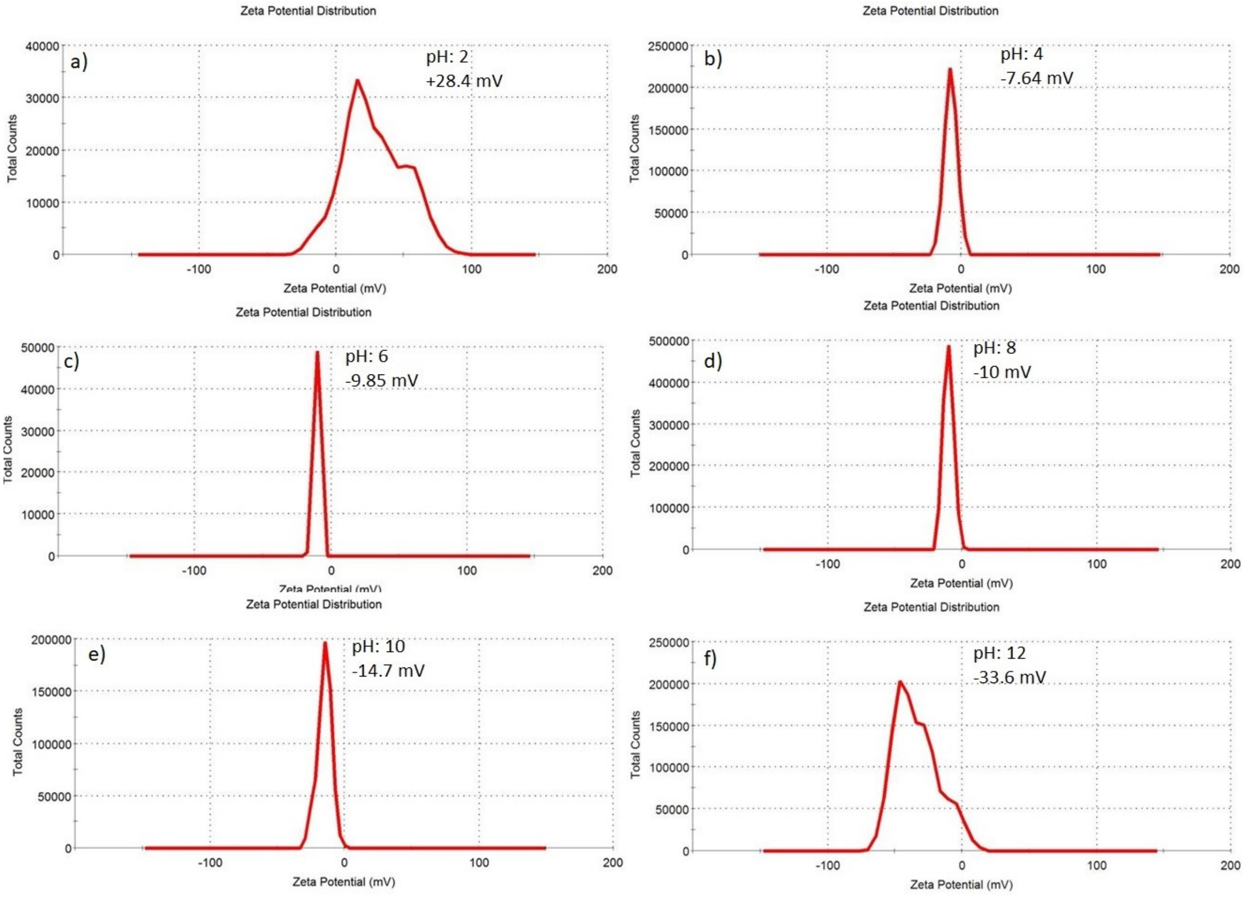

Fig. S2: The zeta potential of as-synthesized ZD at pH 2, 4, 6, 8, 10, and 12.

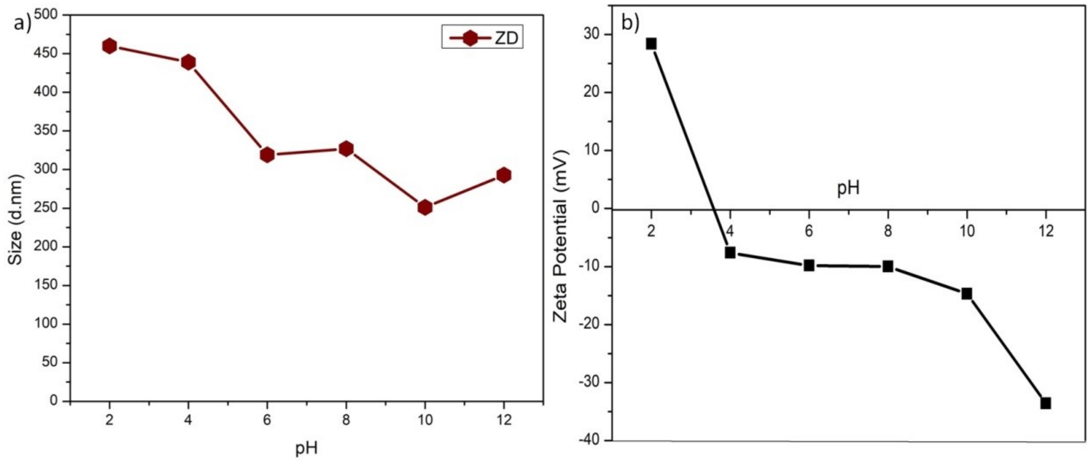

Fig. S3: the variation in (a) hydrodynamic size and (b) zeta potential of as-synthesized ZD at pH 2, 4, 6, 8, 10 and 12.

2.2 Optical Characteristics

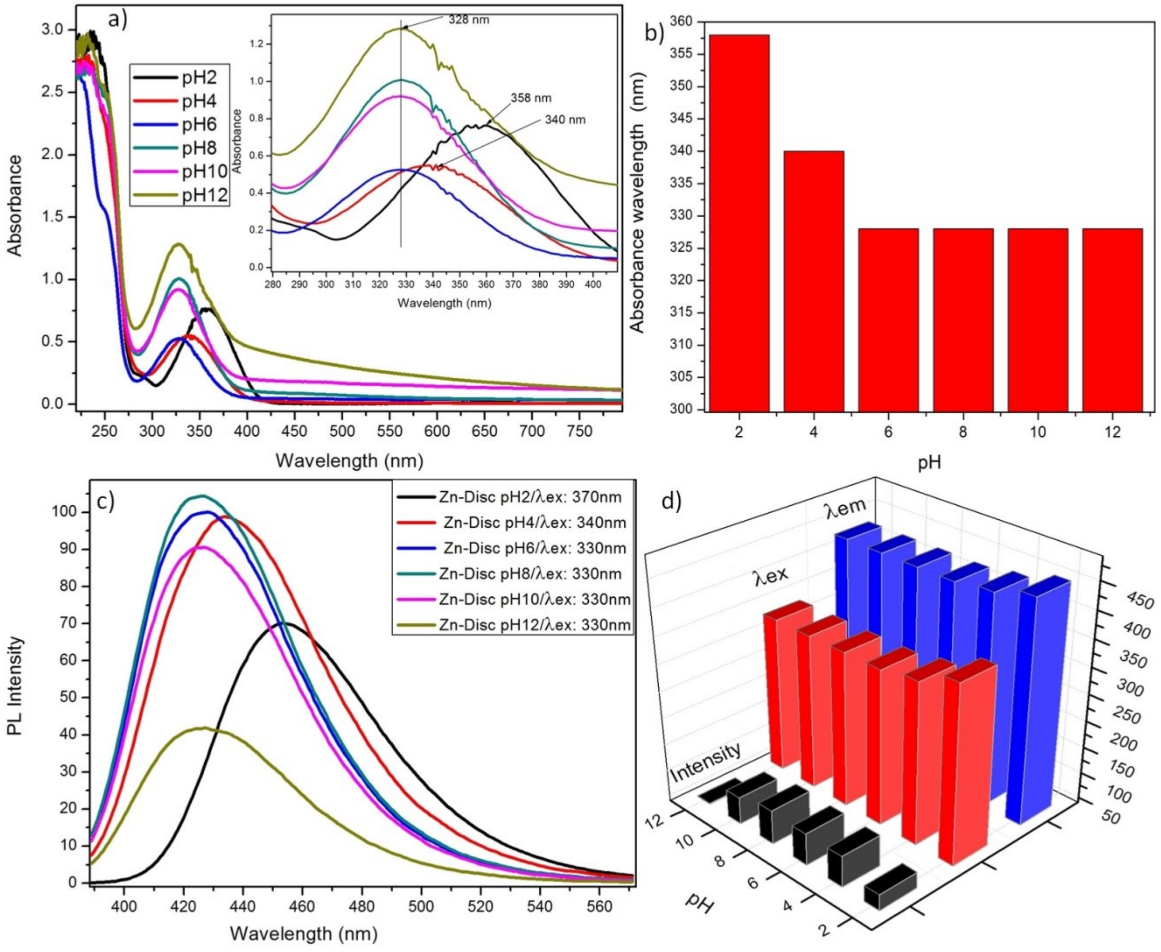

Fig. S4: (a) UV-Vis absorbance spectra, (b) absorbance wavelength, (c) PL emission spectra, and (d) variation in PL emission intensities, excitation wavelength, and emission wavelengths of the as-synthesized ZD at pH 2, 4, 6, 8, 10 and 12.

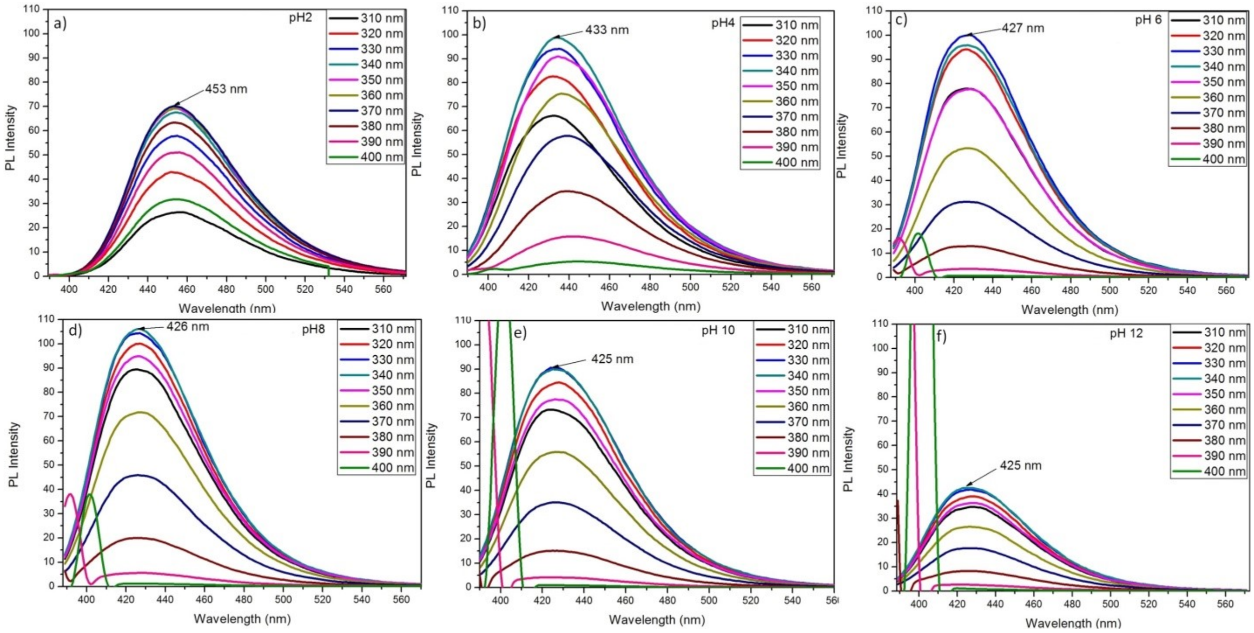

Fig. S5: The PL emission spectra of as-synthesized ZD at pH 2, 4, 6, 8, 10, and 12 at different excitation wavelengths.

2.3 FTIR and RAMAN characterization

Figure S6a presents the FT-IR spectra of MB, ZD and ZD/MB complex. In the FTIR spectrum of ZD, peaks at 3430 and 3300 cm^{-1} correspond to the amino groups while the peaks at 1550 and 1400 cm^{-1}can be ascribed to the asymmetric and symmetric C-O vibrations of carboxylate linker, respectively. Further, the broadband at 1053 cm^{-1} corresponds to C-O stretching vibrations [2]. In the FTIR spectrum of MB, the characteristic peaks appear at 3400, 2920, 1618, 1380, and 1100 cm^{-1} which are assigned to O-H stretching, C-H stretching, N-H bending of amines, and aromatic amines and C-N stretching, respectively. It coincides well with the reported MB spectrum [3]. In the FTIR spectrum of the ZD-MB complex, the peaks at 1630 (N–H bending vibration), 1380 and 1030 cm^{-1} confirmed the adsorption of MB on the ZD surface. It might be due to the H-bonding and electrostatic interaction as supported by the disappearance of the peak at 3300 cm^{-1}(-OH group).

Figure S6b illustrates the Raman spectra of MB, ZD, and ZD-MB complex after the instant addition of dye in ZD. The RAMAN spectrum of MB exhibits the peaks at 1604 cm-^{1} and 1563 cm-^{1} due to C-C stretching: 1486 cm^{-1} from C-N asymmetric stretching and 1395 cm^{-1}by C-H in-plane ring deformation [4]. In the RAMAN spectrum of ZD, the characteristic peak at 1621 cm^{-1} confirmed the presence of functional group >C=O mixed with NH deformation. The multiple peaks ranging from 800- 1000 cm^{-1} denote the multi-substituted aromatic ring of aminobenzene dicarboxylic acid in ZD. The high-intensity peak at 1430 cm^{-1} can be ascribed to CH_2 deformations [5]. Peaks near 696 and 816 cm^{-1} correspond to C-O and C-O-C, respectively. The diminished peak intensity at 1430 and 805- 867 cm^{-1} resulted from the interaction between the MB and ZD particles. The unaltered peak intensity at 1621 cm^{-1} denotes the stable structural bonding even after MB interaction.

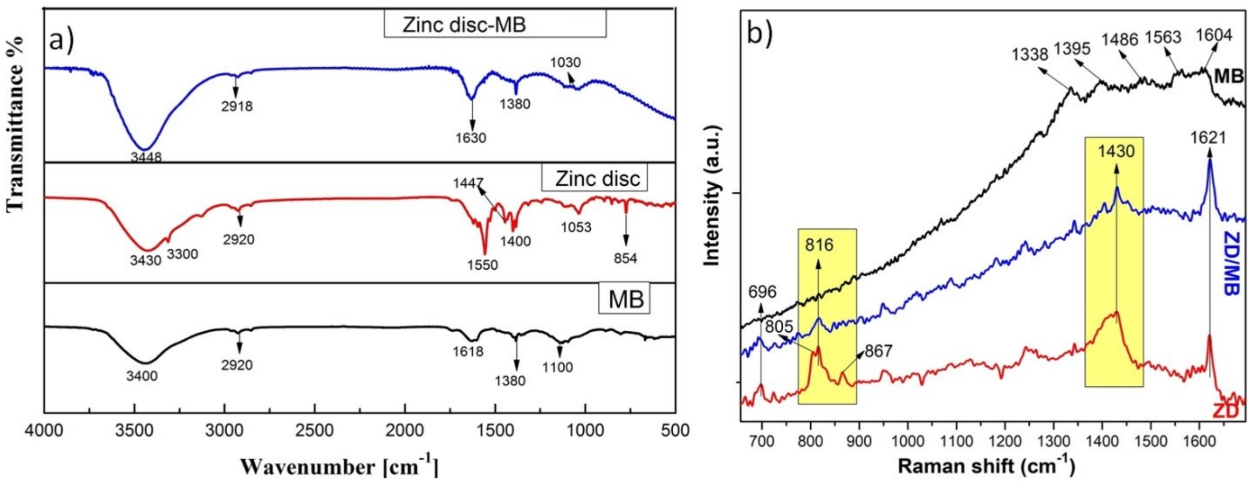

Fig. S6: (a) The FTIR and (b) RAMAN spectra of only MB, only ZD, and ZD/MB complex.

2.4 Surface Characterization

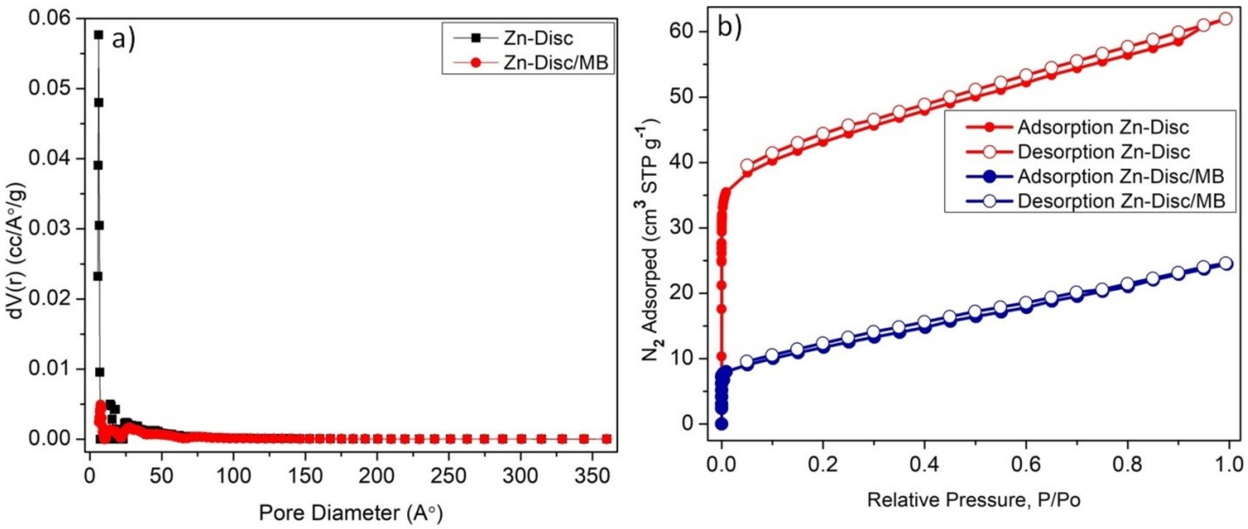

Fig. S7: The BET surface area (a) and pore data of ZD and ZD/MB complex.

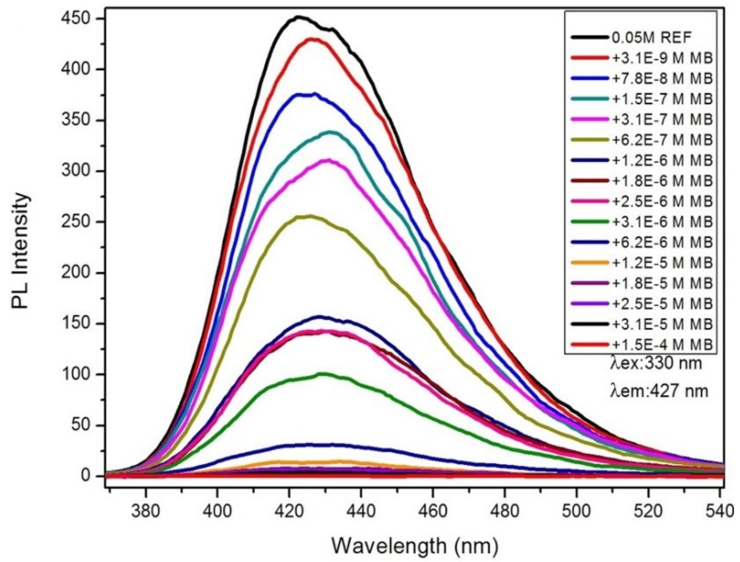

Fig. S8: The variation in PL emission intensity of ZD with increased concentration of MB.

2.5 Selectivity Study for MB sensing

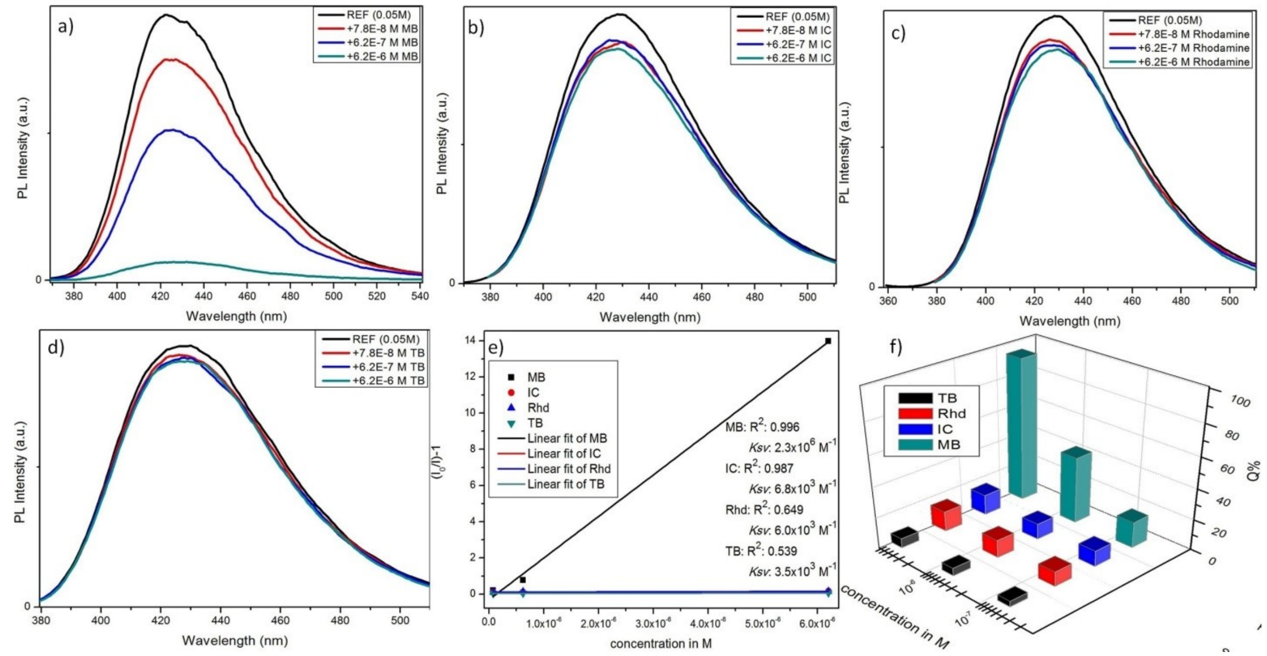

Fig. S9: The variation in PL emission intensity of ZD with increased concentration of (a) MB, (b) IC, (c) RhB, and (d) TB; the (e) Stern-Volmer plots and (f) Q% for the variation in PL emission intensity of ZD in presence pf MB, IC, RhB and TB dyes.

To further strengthen the sensing performance of ZD for MB, the selective activity of this material is also conducted as shown in figure S9. It is evident that the ZD selectively interacts with MB dye but not with any other counterparts such as IC, RhB, and TB. The quenching effect of these dyes was monitored at their three different concentrations i.e., $7.8x10^{-8}$ M, $6.2x10^{-7}$ M, and 6.2x 10^{-6} M. In the presence of 6.2x 10^{-6} M concentration of MB, IC, RhB and TB, the net quenching was attained to be 93.32%,

13.23%, 13.03%, and 6.01% respectively (Fig. S9f). MB resulted in approximately seven times higher than the second-highest quenching that occurred in the presence of IC. This data points to the ultra-selectivity of ZD particles towards MB dye. Furthermore, this outcome was supported by the Stern-Volmer quenching constants of these respective dyes as shown in figure S9e. The *Ksv* of ZD particles for MB, IC, RhB, and TB is calculated to be $2.3x10^{6}$ M^{-1}, $6.8x10^{3}$ M^{-1}, $6x10^{3}$ $M^{-1}$and $3.5x10^{3}$ M^{-1} respectively. It shows that ZD possesses a thousand times higher affinity for MB than that for IC, RhB, and TB dyes. The ultrasensitive and selective performances of ZD particles render them a highly potent sensing material for MB dye detection.

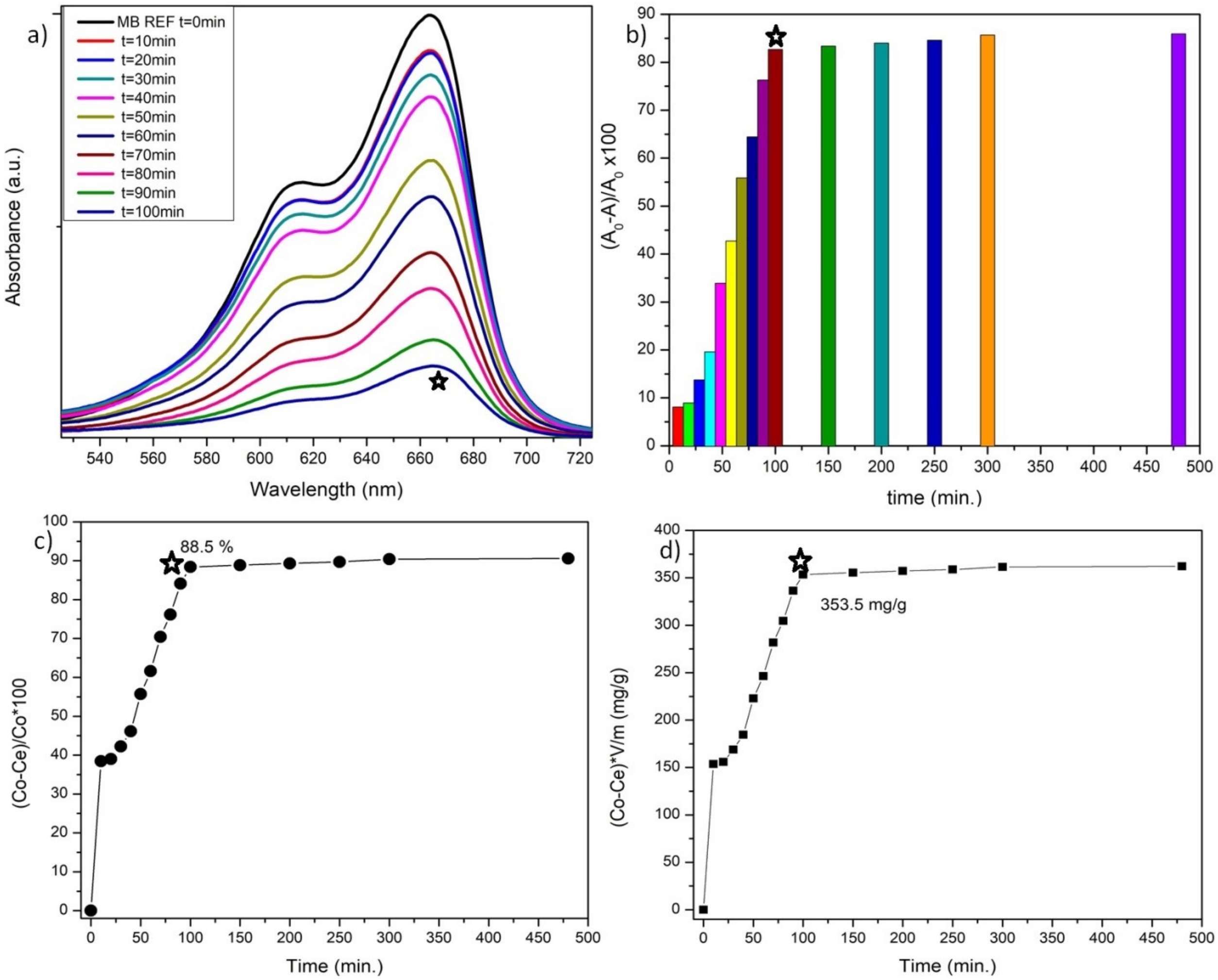

Fig. S10:(a) The variation in the absorbance intensity and (b) the % change in absorption intensity of MB at wavelength 664 nm during adsorption onto ZD concerning the contact time; (c) the removal efficiency and (d) adsorption capacity of ZD for MB adsorption concerning the contact time.

2.6 Photocatalytic degradation of MB in absence of ZD

The photo-degradation activity of ZD for MB dye was executed to assess the photocatalytic efficiency of ZD nanoparticles at pH 8. For reference purposes, only 10 mg/L MB solution (without ZD) was irradiated under UV-illumination for 1 hour at an interval of 5 min (Fig. S11). It can be observed that even after the 1 hr. of irradiation, there is a very negligible change in the UV-absorbance intensity (A) of MB (Fig. S11a). At all the three main SPR band positions i.e., 246 nm, 291 nm, and 663 nm, there is

no notice worthy variation in absorbance (A_0-A), here A_0 and A are the initial and the final absorbance intensities of ZD before and after the UV-irradiation, respectively (Fig. S11b). The full width at half maximum (FWHM) of the absorbance bands of MB at these three wavelengths is minimally affected after instant UV exposure but remains almost unaltered thereafter up to 60 minutes (Fig. S11c).

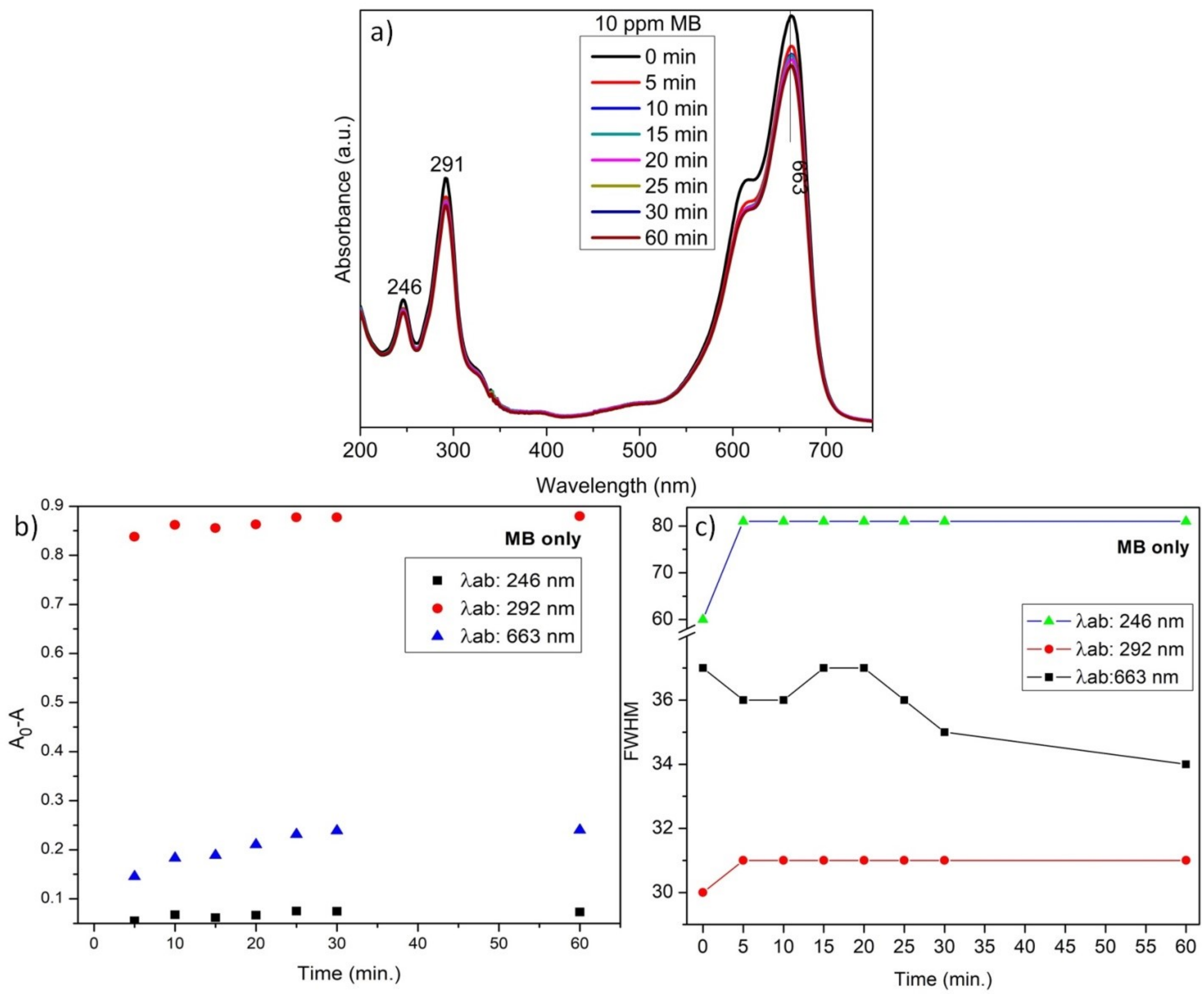

Fig. S11: (a) The variation in the absorbance intensity, (b) the change in the absorption intensity, and (c) the FWHM of only MB at wavelengths 246 nm, 292 nm, and 664 nm concerning the UV-irradiation time for degradation evaluation.

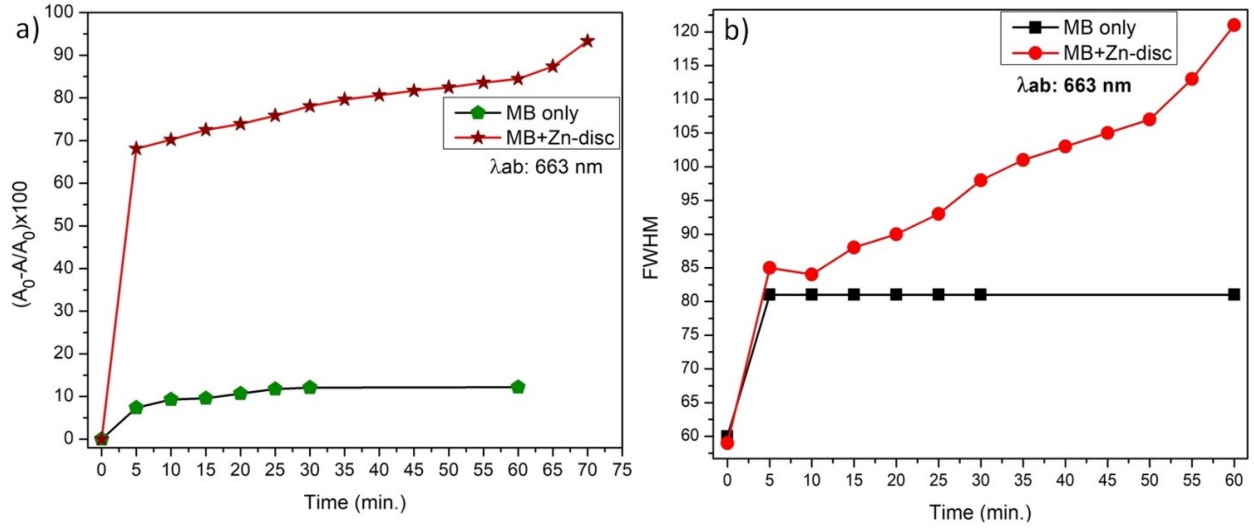

Fig. S12: (a) The % change in absorption intensity, and (b) FWHM of MB at wavelength 663 nm in the absence and presence of ZD concerning the UV-irradiation time for photocatalytic degradation efficiency evaluation.

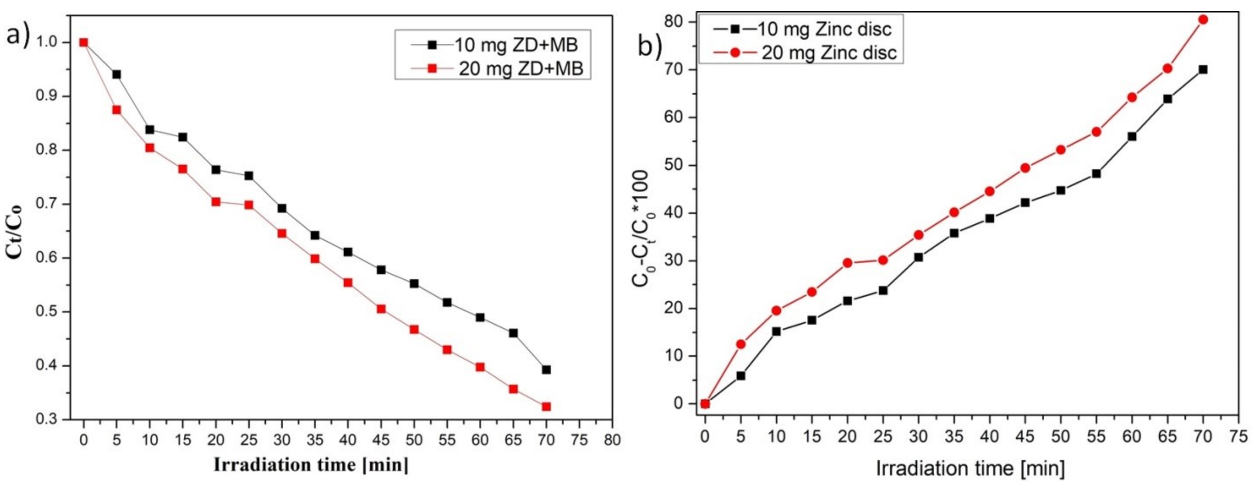

Fig. S13: (a) Variation in MB concentration in presence of 10mg/L and 20 mg/L ZD dosage concerning irradiation time; and (b) Photocatalytic removal efficiency of 10mg/L and 20 mg/L ZD for MB degradation against irradiation time.

2.7 Colorimetric Analysis of Photocatalytic degradation

The colorimetric change in the 10 mg/L MB dye solution irradiated with UV-light in the presence and the absence of ZD catalyst is represented in the pictures shown in figures S14 and S15, respectively. In figure S14, there is a sharp change in the blue color of MB solution treated with ZD at time t=0 min of UV-exposure to greenish color at time t=70 min. But this alteration in MB solution color has not occurred in the absence of ZD even up to 1 hr. of the UV light treatment (Fig. S15).

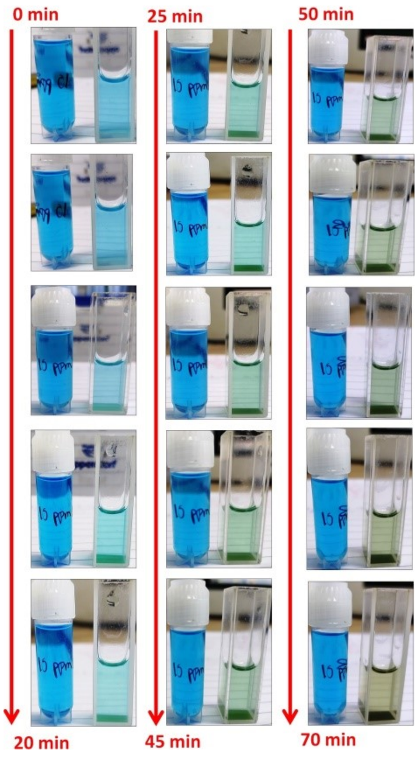

Fig. S14: Photograph of MB reference (left: without UV-irradiation) and ZD/MB complex solution (right: at different time intervals, t= 0, 5, 01, 15, 20, 25, 30, 35, 40, 45, 50, 60, 65 and 70 minutes of UV-irradiation).

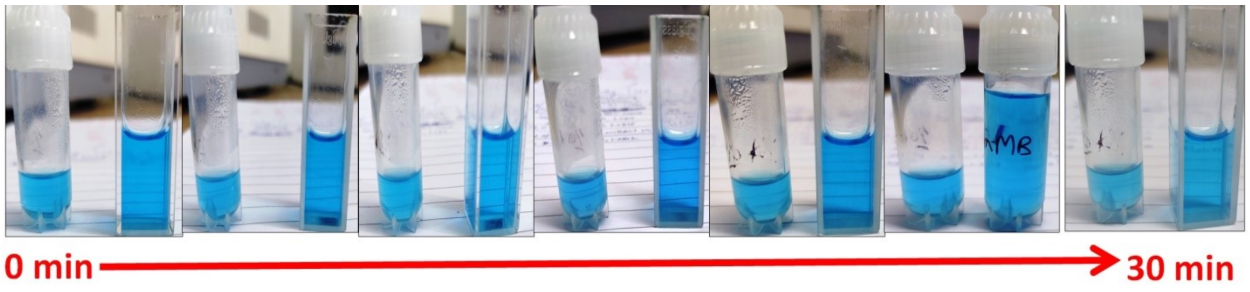

Fig. S15: Photograph of MB solution (left: without UV-irradiation and right: at different time intervals, t= 0, 5, 01, 15, 20, 25, and 30 minutes of UV-irradiation).

2.8 Selectivity Study

To monitor the selective interaction of the ZD particles as adsorbent and photocatalyst for MB dye removal in water, the experiments were also executed with other dyes such as RhB, CV, and IC. The effect of ZD in the respective dye solutions with and without UV light irradiation was investigated using UV-Vis absorption spectroscopy and the data is presented in figure S16.

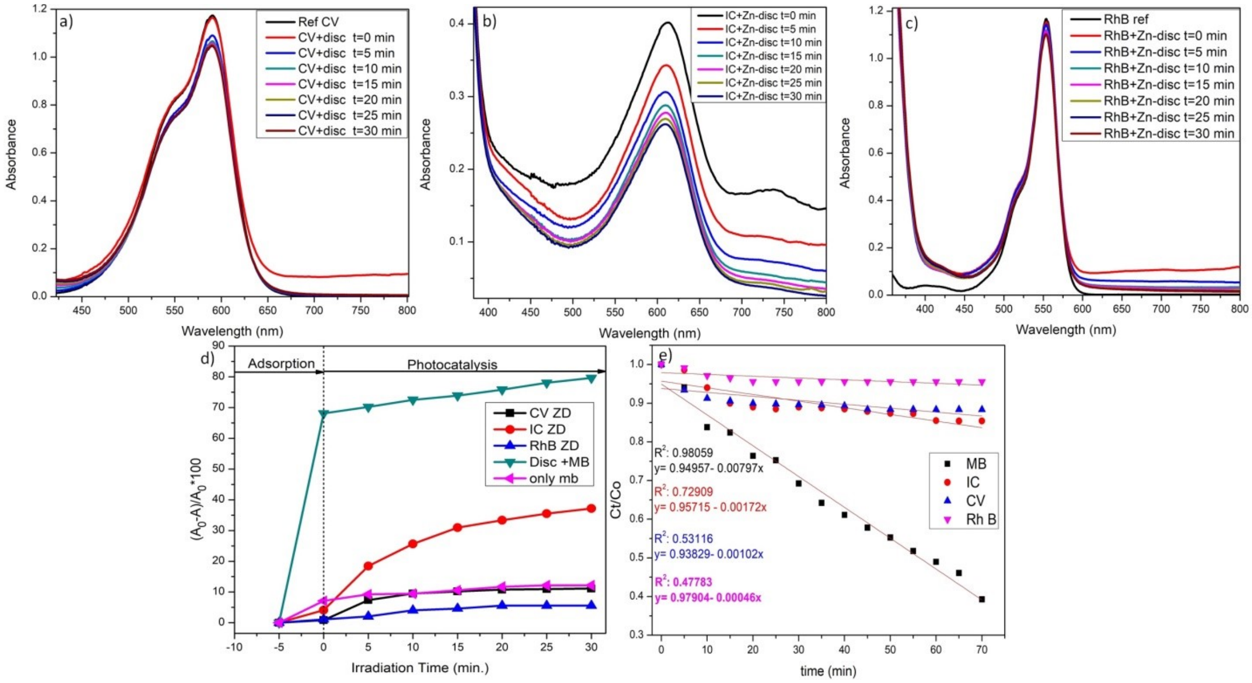

Fig. S16: The variation in the absorbance intensity of (a) CV, (b) IC, and (c) RhB dyes in the presence of ZD concerning the UV-irradiation time; (d) the % change in absorption intensities of CV, IC, RhB and MB dyes in the presence of ZD concerning the UV-irradiation time; and (e) the plot of C_t/C_o versus UV-irradiation time for these dyes in ZD.

It can be noted that there is negligible change in the absorption intensities of CV and RhB dyes in the presence of ZD. The negligible adsorption, as well as photocatalytic degradation effect, was observed in these dyes' concentrations (fig. S16d). Although, IC could have interacted with ZD particles to some extent resulting in noticeable adsorption and a little degradation in the presence of UV light, but after 10 min of UV treatment, the saturation in degradability of IC dye was documented (Fig. S16b). A net 32% of absorption change is noticed even after the UV treatment for 30 minutes which is comparatively very low than that of MB dye (80%) (Fig. S16d). The decrease in the final concentration of MB (R^2= 0.98) in presence of ZD under UV-irradiation for a total period of 70 minutes was very high than that of RhB (R^2= 0.477), CV (R^2= 0.531), and IC (R^2= 0.729) as shown in figure S16e. As studied from literature, Mantasa et. al, 2020, reported that the reason for high adsorption of MB is not only the cationic nature, but also the linearity of the dye [6]. The linear structure of MB enhances the adsorption as it can access the available adsorbent sites more easily. IC also should have made a swift approach to the adsorbent sites due to the linear structure and resulted in its 32% net adsorption. CV being a cationic dye show electrostatic interaction towards the ZD while, the adsorption is declined due to its non-linear and bulky structure in comparison to MB. Therefore, it can be summarized that ZD nanoparticles selectively possess photocatalytic activity for MB degradation in water under UV-light exposure.

2.9 The mechanism of MB dye sensing, adsorption, and photocatalysis degradation

The prominent adsorption mechanisms proposed during adsorptive interplay between the solid ZD-aqueous MB interfaces are represented in the schematic in figure S17. The mechanism of the specific and selective sensing behavior of ZD for MB can be explained by the electrostatic interaction between the lone pairs of amino groups of ZD and MB. The $–NH_2$ group on ZD readily accepts hydrogen proton and acquires positively charged $–NH_3^+$, accelerating the interaction with MB. The types of interactions

that might present between ZD and MB include electrostatic interaction, hydrogen bonding, and π–π stacking. Hydrogen bonding involves polar interaction or extreme dipole-dipole interaction of NH_2 functional group on ZD with complementary groups of MB. Besides, the π–π staking between aromatic rings of both the MB dye and ZD particles is also responsible for the enhancement in the energy transfer between ZD and MB [7]. These interactions ultimately lead to the pore filling of ZD with MB which further results in the adsorption of MB out of the contaminated water. ZD possesses the negatively charged surface at this experimental pH 8 (Fig. S3b) with water as a dispersant which can easily interplay with oppositely charged cationic MB dye.

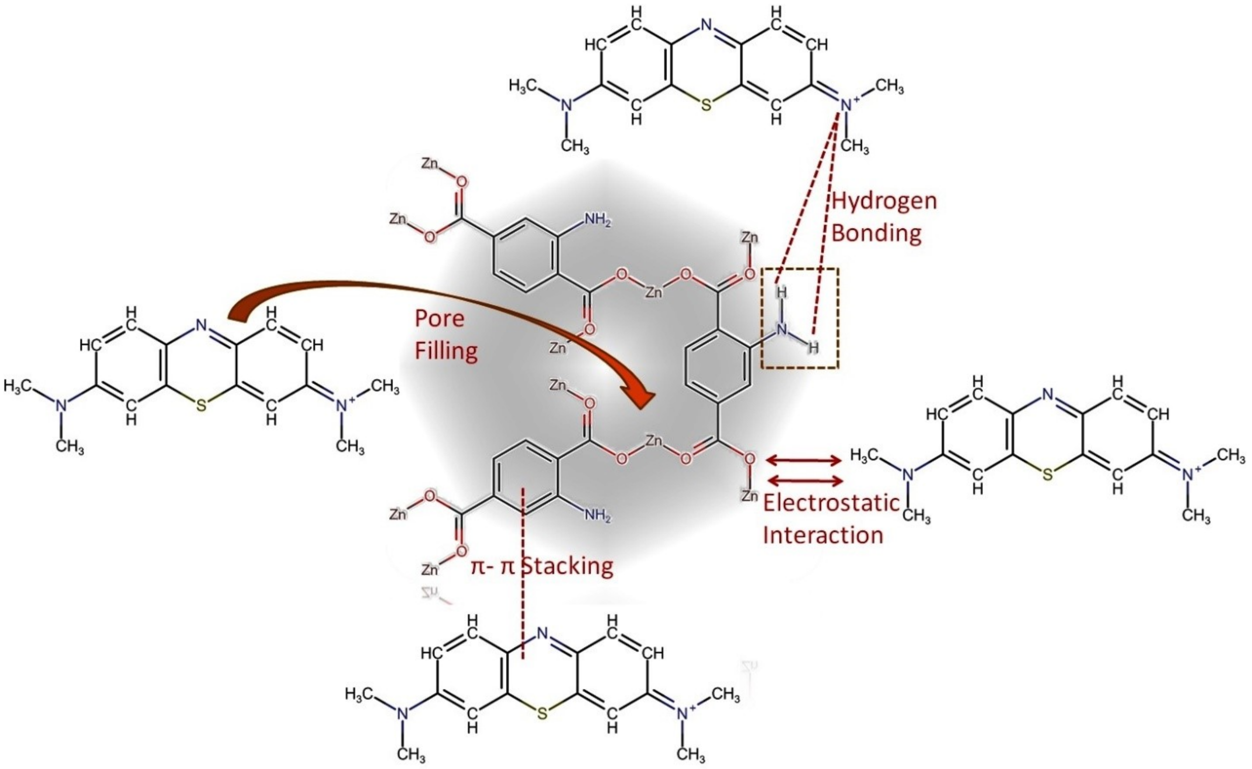

Fig. S17: The schematic representation of MB adsorption mechanism onto ZD.

Additionally, the prevalent mechanism of ZD-derived photocatalytic degradation and discoloration of MB dye is established on the semiconductor theory (Fig. S18). When ZD is illuminated with UV light having photons equal to the energy of the band gap, the electronic excitation occurs. The electrons (e^-) are excited from the highest filled orbital (HOMO) to the lowest vacant orbital (LUMO) and generate the electron-hole pairs (e^-/h^+). The holes and electrons are further involved with the formation of reactive charge species due to the photo excitation of ZD nanoparticles. The holes behave as photo-oxidants and participated in the redox reactions to oxidize the MB to CO_2 and H_2O by forming $OH^\bullet$ radicals. The possible mechanism of this photocatalytic degradation activity of ZD can be represented as follow [7]:

$$ZD + hv \rightarrow ZD\,(e_{CB}^- + h_{VB}^+) \quad (1)$$

$$e_{CB}^- + O_2 \rightarrow O_2^{-\cdot} \quad (2)$$

$$O_2^{-\cdot} + H_2O \rightarrow OH^\cdot + OOH^- \quad (3)$$

$$h_{VB}^+ + H_2O \rightarrow OH^\cdot + H^+ \quad (4)$$

$$OH^{\cdot} + MB \rightarrow CO_2 + H_2O(Degraded\ Products) \quad (5)$$

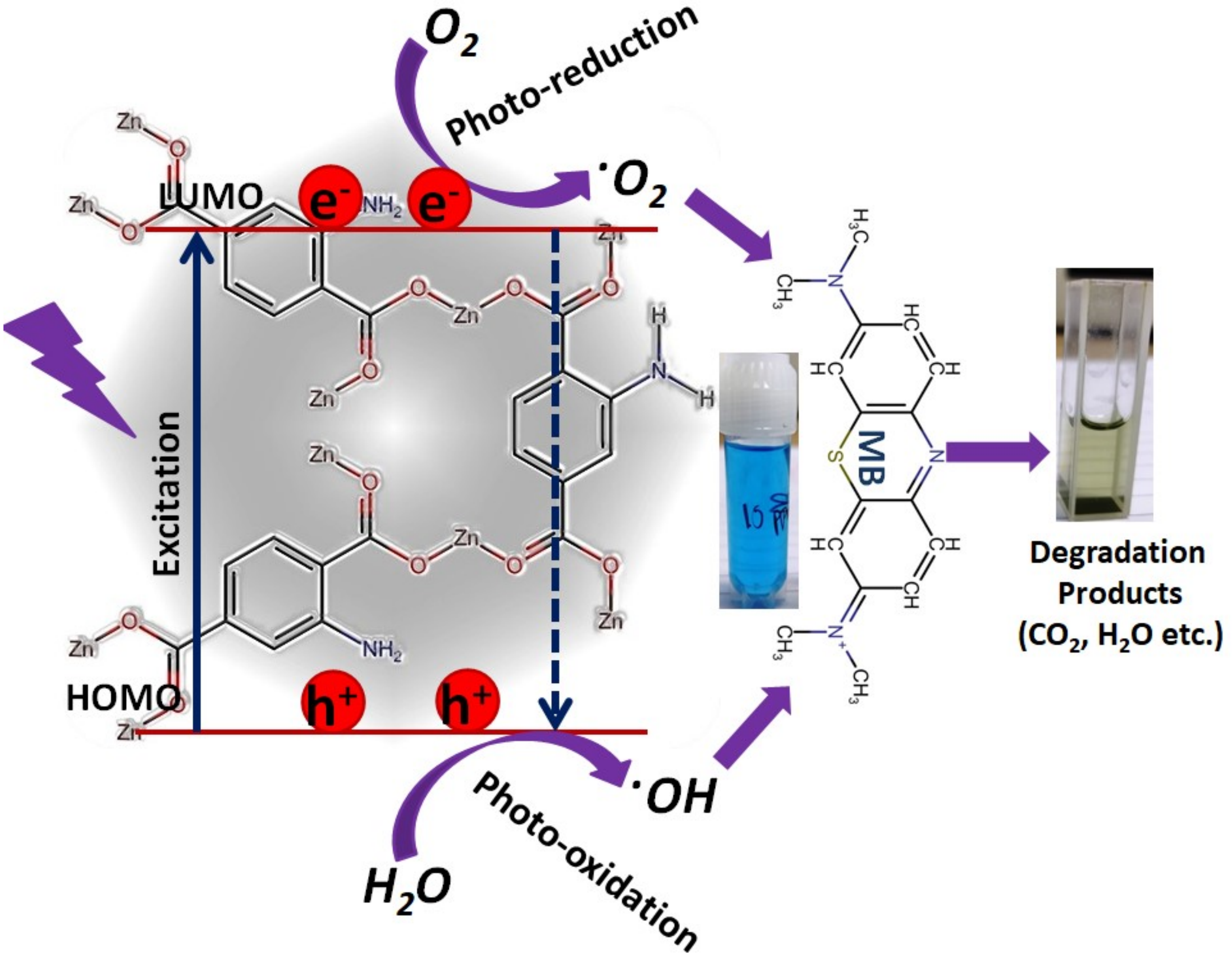

Fig. S18: The schematic representation of ZD mediated photocatalytic degradation of MB.

2.10 Recovery and Reusability

The recovery and reusability of an adsorption or degradation system is a crucial factor for its real field applicability and industrial setups. As explained in hydrodynamic size and surface potential studies, that there are elevated electrostatic interactions and alkali mediated interference resulting in MB degradation. According to this finding, the ZD was regenerated by easy degradation of MB at high pH [1]. It is evident from figure S19a, the maximum decrement in the absorption intensity of MB (10 mg/L) at 664 nm occurred at pH 12 as compared to pH 2 or 8. The adsorption capacity and removal efficiency of ZD; and the %variation in absorbance intensity of MB dye at pH 2, 8, and 12 are shown in figure S19b, and parameters are summarized in table S8. The highest adsorption capacity (377.6 mg/g) and removal efficiency (94.4 %) was achieved by ZD at pH 12. Also, the maximum % change in absorption intensity (88.04 %) was achieved in this alkaline condition. This trend might have occurred due to the produced ·OH (hydroxyl radicals) that electrophilically attacked the cleavage of C-S$^+$=C of MB in alkaline conditions which can be represented as follows [8]:

$$R{-}S^{+} = R + {\cdot}OH \rightarrow R{-}S(=O){-}R + H^{+} \quad (6)$$

Besides, the dispersed dye is cationic (basic type) in nature and gets easily dissolved in organic media having a low dielectric constant [9]. This desorption was achieved by breaking the electrostatic interaction between dye molecules and reactive sites of ZD adsorbent in the presence of NaOH. While at acidic pH, the positively charged ZD surface repelled this cationic dye, which completely justifies the low rate of MB removal by ZD at acidic pH. With the increase in pH, the negative charge of the ZD surface is increased, hence, more attractive forces occur between the photocatalyst and the dye in aqueous solutions, leading to a higher MB removal. Additionally, the hypochromic shift in the absorption wavelength at pH 12 indicates the structural degeneration of MB. Therefore, it can be concluded that the higher pH (alkaline) was most effective for MB removal from ZD. This mechanism of pH-mediated behavior of ZD and MB interaction is represented in the scheme in figure S20.

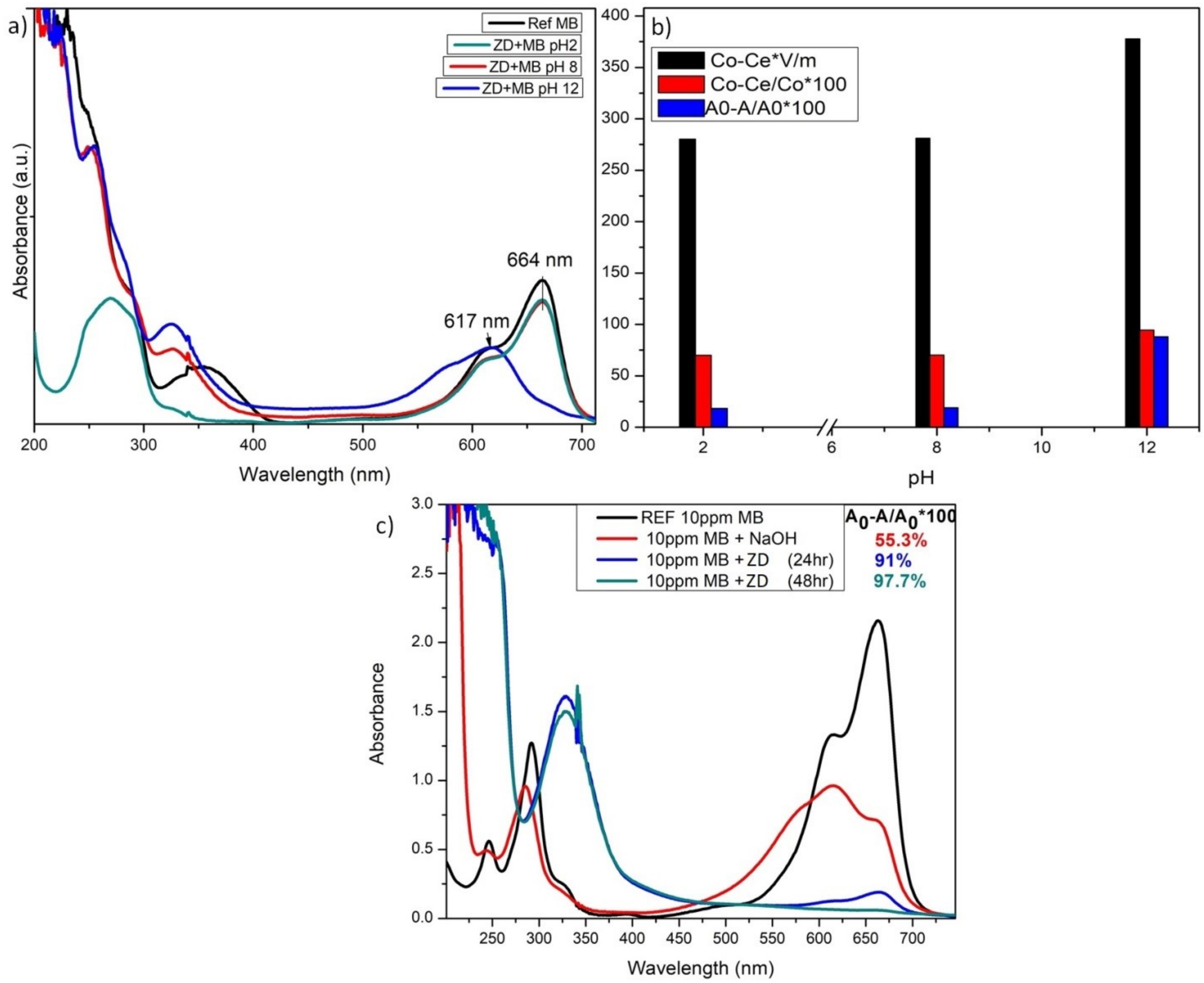

Fig. S19: (a) The UV-Vis absorption spectra of ZD/MB complex; and (b) adsorption capacities of ZD, removal efficiencies of ZD, and % change in absorption intensities of ZD/MB complex at pH 2, 8, 12; (c) The UV-Vis absorption spectra of MB in presence of NaOH and ZD for MB (after 24 hr and 48 hr).

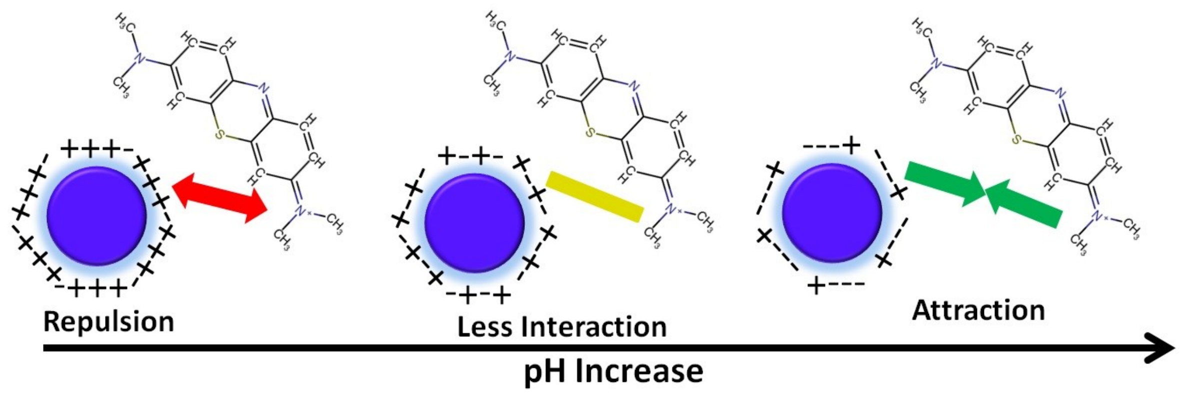

Fig. S20: The schematic representation of ZD/MB interaction at different pH (acidic to alkaline).

These findings are further utilized for the degradation of MB from ZD/MB complex to regenerate the ZD afresh. It can be observed from figure S19c that approximately 55.3 % Mb dye was degraded in the presence of only NaOH. While in the presence of ZD, NaOH was utilized to degrade up to 91 % within 24 hrs which is further increased up to approx. 98% in the next 24 hrs. This data confirms the complete restoration of ZD from the ZD/MB complex by treating it with NaOH solution (Fig. S19c). After this highly commendable MB degradation, the ZD material was centrifuged and collected in a pellet by removing the degenerated MB dye solution in the supernatant.

Thereafter, this collected ZD was characterized using FTIR and RAMAN spectroscopies for any alteration in its functional structure as shown in figures S21a and S21b respectively. It can be noticed that the characteristic FTIR peaks of recovered ZD remain unaffected and completely match with the bare ZD spectrum as represented in figure S6a. The RAMAN spectrum of these recovered-ZD particles is also completely restored as to the bare ZD shown in figure S6b. Therefore, these findings describe the successful degradation of MB dye from the ZD/MB complex and the successful recycling of the ZD with well retained functional characteristics. Further, this recovered ZD was reused for MB adsorption as shown in figure S21c. It can be observed the empty sites of recovered ZD successfully reabsorbed the MB out of the contaminated water sample. The characteristic absorption band of MB at 664 nm confirms the presence of MB onto the reused ZD. To strengthen the reusability of ZD, the recovery study was conducted in four cycles for MB adsorption onto ZD (Fig. S21d). Although, the percentage recovery decreased continuously with successive cycles yet was brilliant enough as even in the fourth cycle, the best desorption capacity was found to be 58% for MB dye.

Therefore, this study well verse about the applicability of zinc-disc nanomaterial as an excellent sensor, adsorbent, and photocatalyst with high recovery and reusability. It can be suggested that these luminescent, porous, and readily to be functionalized ZD nanoparticles can be applied for various other applications such as sensing, cell-imaging, drug delivery, adsorption, removal of various other organic/inorganic contaminants, etc.

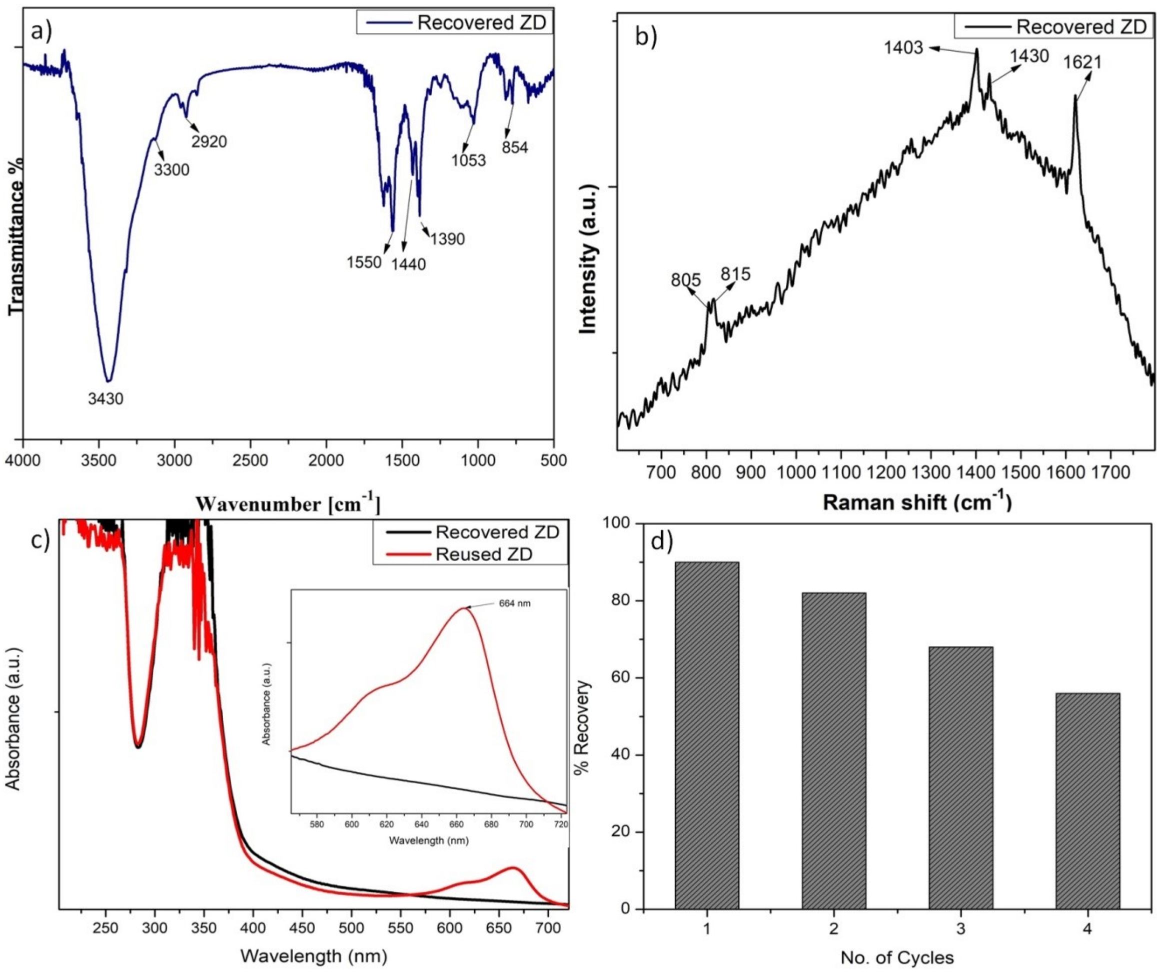

Fig. S21: (a) FTIR and (b) RAMAN spectra of recovered ZD; (c) the absorbance spectra of recovered and reused ZD for MB presence; and (d) the % recovery assessment of ZD up to four cycles.

Table S1: The summarized XRD parameters for ZD.

S.No	2θ	d-spacing	FWHM	Hkl
**1**	38.2	2.35	0.2160	111
**2**	44.4	2.03	0.1680	200
**3**	64.8	1.43	0.3840	220
**4**	77.8	1.22	0.2400	311

Table S2: The surface area data of ZD and ZD/MB

Property	ZD	ZD/MB
MultiPoint BET	1.598e+02 m²/g	4.113e+01 m²/g
BJH method cumulative adsorption surface area	2.197e+01 m²/g	1.585e+01 m²/g
BJH method cumulative desorption surface area	1.867e+01 m²/g	1.264e+01 m²/g
DH method cumulative adsorption surface area	2.251e+01 m²/g	1.624e+01 m²/g
DH method cumulative desorption surface area	1.911e+01 m²/g	1.294e+01 m²/g
DFT cumulative surface area	1.994e+02 m²/g	3.703e+01 m²/g

Table S3: The pore volume data of ZD and ZD/MB

Property	ZD	ZD/MB
Total pore volume for pores with Radius less than 1963.17 Å at P/Po = 0.995	9.592e-02 cc/g	3.781e-02 cc/g
BJH method cumulative adsorption pore volume	3.388e-02 cc/g	2.357e-02 cc/g
BJH method cumulative desorption pore volume	2.970e-02 cc/g	2.023e-02 cc/g
DH method cumulative adsorption pore volume	3.333e-02 cc/g	2.320e-02 cc/g
DH method cumulative desorption pore volume	2.923e-02 cc/g	1.992e-02 cc/g
DFT method cumulative pore volume	8.811e-02 cc/g	3.498e-02 cc/g

Table S4: The data of equilibrium models studied for adsorption

Model	Equation	Parameters	
		q_m (mg g^{-1})	207.0
**Langmuir**	$\frac{C_e}{q_e}=\frac{1}{k_L q_m}+\frac{C_e}{q_m}$	K_L (L mg^{-1})	0.2445
		R_L	0.039
		R^2	**0.9890**
Freundlich		K_F (mg g^{-1})	1.49

	$\ln q_e = \frac{1}{n}.\ln c_e + \ln k_F$	n	5.15
		R^2	0.8772
Temkin	$\ln q_e = \frac{1}{n}.\ln c_e + \ln k_F$	K_T (L mg^{-1})	7.725
		RT/b_T (kJ/mol)	31.09
		R^2	0.8787

Table S5: Kinetic parameters for the adsorption of MB onto ZD

Model	Linear Equation	Parameters	
First Order	$\frac{1}{q_t} = \frac{1}{q_t} + \frac{k_1}{q_e t}$	k_1 (min^{-1})	16.12
		$q_{e,calc}$ (mg g^{-1})	337.83
		R^2	0.69836
Second Order	$\frac{1}{C_e} - \frac{1}{C_0} = k_2 t$	k_2 (gmg^{-1} min^{-1})	0.00237
		R^2	0.68534
**Pseudo-Second Order**	$\frac{t}{q_t} = \frac{1}{k_2 {q_e}^2} + \frac{t}{q_e}$	k_2 (gmg^{-1} min^{-1})	0.00011
		$q_{e,calc}$ (mg g^{-1})	386.1
		R^2	**0.99**
Elovich	$q_t = \frac{In\ (a * b)}{b} + \frac{Int}{b}$	a (mg g^{-1} min^{-1})	124.74
		b (gmg^{-1})	0.0137
		R^2	0.83135
Intraparticle Diffusion	$q_t = k_p t^{1/2} + c$	k_p (mg g^{-1} $min^{-1/2}$)	13.45
		c	143.52
		R^2	0.6861

Table S6: Comparison of the maximum uptake/ adsorption of MB onto ZD with that of various adsorbents

S. No.	Adsorbent	q_{max} (mg g^{-1})	Reference
**1**	Cedar cone	4.55	[10]
**2**	Baker's yeast	25	[11]
**3**	Jute stick powder	87.7	[12]
**4**	Kaolinite	46.08	[13]
**5**	Acid-treated dika nut	232	[14]
**6**	Fly ash	10	[15]
**7**	Natural coal	40.82	[16]

**8**	Microwave-treated nilotica leaf	24.39	[17]
**9**	Sugarcane baggas	51.5	[18]
**10**	Modified coir pit	14.9	[19]
**11**	HM-MIL-101	21.0	[20]
**12**	MIL-100 (Fe)	736.2	[20]
**13**	MIL-100 (Cr)	645.3	[21]
**14**	MOF-235	252.0	[22]
**15**	Zinc-disc	386.1	**This work**

Table S7: Kinetic parameters for the photocatalytic degradation of MB with ZD nanocatalyst

**Model**	**Linear plot scale parameters**	**Parameters**		
			10 mg/L ZD	20 mg/L ZD
**Pseudo Zero Order**	C (mg L^{-1}) *vs* t (min.)	k ($mgL^{-1}min^{-1}$)	0.02566	0.008141
		R^2	0.93151	0.98151
**Pseudo First Order**	-ln C_t/C_o *vs* t (min.)	k ($min^{-1)}$)	0.015	0.011
		R^2	**0.99**	0.989
**Pseudo-Second Order**	1/Ct (L mg^{-1}) *vs* t (min.)	k (Lmg^{-1} min^{-1})	0.03488	0.00969
		R^2	0.90681	0.93303

Table S8: The comparison of adsorption capacities, removal efficiencies and absorption intensity change of ZD for MB at various pH

**pH**	**Co-Ce*v/m**	**Co-Ce/Co*100**	**A_0-A/A_0*100**
**2**	280.14792	70.03698	18.42308
**8**	281.06367	70.26592	19.07692
**12**	377.65898	94.41474	88.04808

References:

1. M. Zamouche and O. Hamdaoui, *Energy Procedia*, 2012,**18**, 1228.
2. S. Devi, S. Shaswat, V. Kumar, A. Sachdev, P. Gopinath, and S. Tyagi, *Microchimica Acta*, 2020, **187**, 1-10. https://doi.org/10.1007/s00604-020-04496-0
3. D.L. Pavia, G.M. Lampman, G.S. Kriz and J.A. Vyvyan, *Introduction to spectroscopy Cengage learning*, 2014.

4. M. del Pilar Rodríguez-Torres, L.A. Díaz-Torres and S. Romero-Servin, *International Journal of Molecular Sciences*, 2014, **15**, 19239. https://doi.org/10.3390/ijms151019239
5. D.Y. Siberio-Pérez, A.G. Wong-Foy, O.M. Yaghi and A.J. Matzger, *Chemistry of materials*, 2007, **19**, 3681. https://doi.org/10.1021/cm070542g
6. I. Mantasha, H.A. Saleh, K.M. Qasem, M. Shahid, M. Mehtab and M. Ahmad, *Inorganica Chimica Acta*, 2020, **511**, 119787. https://doi.org/10.1016/j.ica.2020.119787
7. C. Van Tran, D.D. La, P.N.T. Hoai, H.D. Ninh, P.N.T. Hong, T.H.T. Vu, A.K. Nadda, X.C. Nguyen, D.D. Nguyen and H.H. Ngo, *Journal of hazardous materials*, 2021, **420**, 126636. https://doi.org/10.1016/j.jhazmat.2021.126636
8. T.A. Kurniawan, Z. Mengting, D. Fu, S.K. Yeap, M.H.D. Othman, R. Avtar and T. Ouyang, *Journal of environmental management*, 2020, **270**, 110871. https://doi.org/10.1016/j.jenvman.2020.110871
9. S.P. Shukla and N. Dhiman, *Environmental Earth Sciences*, 2017, **76**, 1-12. https://doi.org/10.1007/s12665-017-7030-x
10. J.T. da Fontoura, G.S. Rolim, B. Mella, M. Farenzena and M. Gutterres, *Journal of environmental chemical engineering*, 2017, **5**, 5076. https://doi.org/10.1016/j.egypro.2012.05.138
11. J.X. Yu, B.H. Li, X.M. Sun, Y. Jun and R.A. Chi, *Biochemical Engineering Journal*, 2009, 45, **145**. https://doi.org/10.1016/j.bej.2009.03.007
12. G.C. Panda, S.K. Das, and A.K. Guha, *Journal of Hazardous Materials*, 2009, **164**, 374. https://doi.org/10.1016/j.jhazmat.2008.08.015
13. T.A. Khan, S. Dahiya, and I. Ali, *Applied Clay Science*, 2012, **69**, 58. https://doi.org/10.1016/j.clay.2012.09.001
14. Z. Tong, P. Zheng, B. Bai, H Wang, and Y. Suo, *Catalysts*, 2016, **6**, 58. https://doi.org/10.3390/catal6040058
15. S.H. Chang, K.S. Wang, H.C. Li, M.Y. Wey and J.D. Chou, *Journal of hazardous materials,* 2009, **172**, 1131. https://doi.org/10.1016/j.jhazmat.2009.07.106
16. N. Hasani, T. Selimi, A. Mele, V. Thaçi, J. Halili, A. Berisha and M. Sadiku, *Molecules,* 2022, **27**, 1856. https://doi.org/10.3390/molecules27061856
17. T. Santhi, A.L. Prasad and S. Manonmani, *Arabian Journal of Chemistry*, 2014, **7**, 494. https://doi.org/10.1016/j.arabjc.2010.11.008
18. Z. Zhang, I.M. O'Hara, G.A. Kent and W.O. Doherty, *Industrial Crops and Products*, 2013, **42**, 41. https://doi.org/10.1016/j.indcrop.2012.05.008
19. M.V. Sureshkumar and C. Namasivayam, *Colloids and Surfaces A: Physicochemical and Engineering Aspects,* 2008, **317**, 277. https://doi.org/10.1016/j.colsurfa.2007.10.026
20. X.X. Huang, L.G. Qiu, W. Zhang, Y.P. Yuan, X. Jiang, A.J. Xie, Y.H. Shen and J.F. Zhu, *CrystEngComm,* 2012, **14**, 1613.https://doi.org/10.1039/C1CE06138K
21. M. Tong, D. Liu, Q. Yang, S. Devautour-Vinot, G. Maurin and C. Zhong, *Journal of Materials Chemistry A*, 2013, **1**, 8534. https://doi.org/10.1039/C3TA11807J
22. E. Haque, J.W. Junand and S.H. Jhung, *Journal of Hazardous materials*, 2011, **185**, 507. https://doi.org/10.1016/j.jhazmat.2010.09.035

Supplementary figure captions:

Fig. S1: The hydrodynamic size of as-synthesized ZD at pH 2, 4, 6, 8, 10, and 12.

Fig. S2: The zeta potential of as-synthesized ZD at pH 2, 4, 6, 8, 10, and 12.

Fig. S3: The variation in (a) hydrodynamic size and (b) zeta potential of as-synthesized ZD at pH 2, 4, 6, 8, 10 and 12.

Fig. S4: (a) UV-Vis absorbance spectra, (b) absorbance wavelength, (c) PL emission spectra, and (d) variation in PL emission intensities, excitation wavelength, and emission wavelengths of the as-synthesized ZD at pH 2, 4, 6, 8, 10 and 12.

Fig. S5: The PL emission spectra of as-synthesized ZD at pH 2, 4, 6, 8, 10, and 12 at different excitation wavelengths.

Fig. S6: (a) The FTIR and (b) RAMAN spectra of only MB, only ZD, and ZD/MB complex.

Fig. S7: The BET surface area (a) and pore data of ZD and ZD/MB complex.

Fig. S8: The variation in PL emission intensity of ZD with increased concentration of MB.

Fig. S9: The variation in PL emission intensity of ZD with increased concentration of (a) MB, (b) IC, (c) RhB, and (d) TB; the (e) Stern-Volmer plots and (f) Q% for the variation in PL emission intensity of ZD in presence pf MB, IC, RhB and TB dyes.

Fig. S10:(a) The variation in the absorbance intensity and (b) the % change in absorption intensity of MB at wavelength 664 nm during adsorption onto ZD concerning the contact time; (c) the removal efficiency and (d) adsorption capacity of ZD for MB adsorption concerning the contact time.

Fig. S11: (a) The variation in the absorbance intensity, (b) the change in the absorption intensity, and (c) the FWHM of only MB at wavelengths 246 nm, 292 nm, and 664 nm concerning the UV-irradiation time for degradation evaluation.

Fig. S12: (a) The % change in absorption intensity, and (b) FWHM of MB at wavelength 663 nm in the absence and presence of ZD concerning the UV-irradiation time for photocatalytic degradation efficiency evaluation.

Fig. S13: (a) Variation in MB concentration in presence of 10mg/L and 20 mg/L ZD dosage concerning irradiation time; and (b) Photocatalytic removal efficiency of 10mg/L and 20 mg/L ZD for MB degradation against irradiation time.

Fig. S14: Photograph of MB reference (left: without UV-irradiation) and ZD/MB complex solution (right: at different time intervals, t= 0, 5, 01, 15, 20, 25, 30, 35, 40, 45, 50, 60, 65 and 70 minutes of UV-irradiation).

Fig. S15: Photograph of MB solution (left: without UV-irradiation and right: at different time intervals, t= 0, 5, 01, 15, 20, 25, and 30 minutes of UV-irradiation).

Fig. S16: The variation in the absorbance intensity of (a) CV, (b) IC, and (c) RhB dyes in the presence of ZD concerning the UV-irradiation time; (d) the % change in absorption intensities of CV, IC, RhB and MB dyes in the presence of ZD concerning the UV-irradiation time; and (e) the plot of C_t/C_o versus UV-irradiation time for these dyes in ZD.

Fig. S17: The schematic representation of MB adsorption mechanism onto ZD.

Fig. S18: The schematic representation of ZD mediated photocatalytic degradation of MB.

Fig. S19: (a) The UV-Vis absorption spectra of ZD/MB complex; and (b) adsorption capacities of ZD, removal efficiencies of ZD, and % change in absorption intensities of ZD/MB complex at pH 2, 8, 12; (c) The UV-Vis absorption spectra of MB in presence of NaOH and ZD for MB (after 24 hr and 48 hr).

Fig. S20: The schematic representation of ZD/MB interaction at different pH (acidic to alkaline).

Fig. S21: (a) FTIR and (b) RAMAN spectra of recovered ZD; (c) the absorbance spectra of recovered and reused ZD for MB presence; and (d) the % recovery assessment of ZD up to four cycles.

Table Captions:

Table S1: The summarized XRD parameters for ZD.

Table S2: The surface area data of ZD and ZD/MB.

Table S3: The pore volume data of ZD and ZD/MB.

Table S4: The data of equilibrium models studied for adsorption.

Table S5: Kinetic parameters for the adsorption of MB onto ZD.

Table S6: Comparison of the maximum uptake/ adsorption of MB onto ZD with that of various adsorbents.
Table S7: Kinetic parameters for the photocatalytic degradation of MB with ZD nanocatalyst.
Table S8: The comparison of adsorption capacities, removal efficiencies, and absorption intensity change of ZD for MB at various pHs.
